# Substance P receptor signaling contributes to host maladaptive responses during enteric bacterial infection

**DOI:** 10.1073/pnas.2415287122

**Published:** 2025-02-12

**Authors:** Michael Cremin, Valerie T. Ramirez, Kristina Sanchez, Emmy Tay, Kaitlin Murray, Ingrid Brust-Mascher, Colin Reardon

**Affiliations:** ^a^Department of Anatomy, Physiology and Cell Biology, School of Veterinary Medicine, University of California Davis, Davis, CA 95616

**Keywords:** neuroimmunology, mucosal Immunology, inflammation, *Citrobacter rodentium*, immunopathology

## Abstract

Immune responses in the intestine are intricately balanced to prevent pathogen entry without inducing immunopathology. The nervous system is well established to interface with the immune system to fine-tune immunity in various organ systems including the gastrointestinal tract. Specialized sensory neurons can detect bacteria, bacterial products, and the resulting inflammation, to coordinate the immune response in the gastrointestinal tract. These sensory neurons release peptide neurotransmitters such as Substance P (SP), to induce both neuronal signaling and localized responses in nonneuronal cells. With this in mind, we assessed the immunoregulatory roles of SP receptor signaling during enteric bacterial infection with the noninvasive pathogen *Citrobacter rodentium*. Pharmacological antagonism of the SP receptor significantly reduced bacterial burden and prevented colonic crypt hyperplasia. Mice with SP receptor signaling blockade had significantly reduced inflammation and recruitment of T cells in the colon. Reduced colonic T cell recruitment is due to reduced expression of adhesion molecules on colonic endothelial cells in SP receptor antagonist-treated mice. Using SP receptor T cell conditional knockout mice, we further confirmed SP receptor signaling enhanced select aspects of T cell responses. Our data demonstrate that SP receptor signaling can significantly reduce inflammation and prevent host-maladaptive responses without impinging upon host protection.

## Significance

Control of intestinal inflammation is a critical determinant to well-being. Immune reactions to diet, components of the microbiota or otherwise innocuous substances in the lumen can trigger significant disease or immunopathology. Despite this requirement, as a mucosal surface, the immune system must also respond to and prevent pathogens from gaining entry to the host. Here, we identify that the neurotransmitter receptor for Substance P plays a critical role in this process, with antagonism of the receptor reducing enteric infection-induced immunopathology.

Control of immune responses in the gastrointestinal tract is a critical determinant of health. This mucosal surface is exposed to a variety of antigens derived from commensal microorganisms; however, it can also be an entry point for pathogens. Perhaps unsurprisingly, a variety of overlapping regulatory mechanisms have evolved that allow for robust host protection, while limiting inappropriate responses to otherwise innocuous substances. Communication between the nervous and immune systems is now well established to regulate immune cell function and consequently immunological outcomes in several organ systems including the intestinal tract (1–4). These neuroimmune interactions are aided by the colon’s dense innervation, with neurons that reside within (intrinsic) and neurons with cell bodies that are outside (extrinsic) but project into discrete portions of the intestine. This intrinsic innervation forms the enteric nervous system (ENS) that regulates many aspects of intestinal physiology (5, 6). Extrinsic innervation encompasses highly specialized sensory neurons that express the polymodal nociceptive receptor TRPV1, and as such can be activated by noxious stimuli such as extreme heat, danger-associated molecular patterns, bacterial components, and immune mediators produced as a consequence of inflammation (1, 7, 8).

Far from simple detection of inflammation, sensory neurons are required to coordinate host protection during enteric bacterial infection. Activation of these neurons not only can induce classical reflex arc where ascending signals are coordinated in the dorsal root ganglia to drive efferent neuronal activity but can cause localized release of neurotransmitters. During *Salmonella typhimurium* infection of the small intestine, release of the neuropeptide calcitonin gene-related peptide (CGRP) was found to exert a host-protective role (9). Similarly, we have previously demonstrated that mice with targeted depletion of peripheral sensory afferent neurons (10), or TRPV1 knockout (KO) mice (11), experienced increased bacterial burden and enhanced colonic pathology during *Citrobacter rodentium* infection. Although our studies suggested that CGRP did not exert a host-protective role, the neuronally derived signal that coordinated host responses during enteric bacterial infection with this pathogen was not elucidated. Upon activation, nociceptive neurons release several immunologically relevant neurotransmitters in addition to CGRP, such as Substance P (SP). This neurotransmitter has long been described to not only be the mediator encoding pain but has immune effects as well (1, 2, 12, 13). In the intestine, release of SP from the ENS is well appreciated to exert secretomotor function as an excitatory neurotransmitter (14). Additionally, localized release of SP is the basis for neurogenic inflammation, where this peptide acts as a chemoattractant that can activate blood endothelial cells (BEC), causing increased expression of adhesion molecules and vascular permeability (15, 16). It is critical to note that these physiological effects are due to activation of the SP receptor where three ligands have been identified to induce receptor activation. These include not only SP and Neurokinin A, encoded by the preprotachykinin A/Tac1 gene, but also hemokinin-1, encoded by the Tac4 gene (17–20). In the intestinal tract, SP signaling through the SP receptor (TACR1) has a proinflammatory effect. During acute induced models of colitis, deficiency in SP, or prior ablation of sensory afferent neurons, reduced disease severity (21). Similarly, the highly selective SP receptor antagonist CP96345 significantly reduced DSS-induced inflammation in rats (22). Thus, SP receptor signaling can increase host-maladaptive immune responses in vivo resulting in immunopathology. Highlighting the complexity of neuroimmune responses and in contrast to SP receptor signaling driving host-maladaptive responses in colitis, this receptor appears to be critical to the host response during bacterial or parasitic infection. Infection with the invasive enteric bacterial pathogen *S. typhimurium* was significantly worsened with SP receptor antagonists due to reduced Th1 immune responses and mortality. This dichotomous role of SP receptor signaling suggests it has a unique role in the control of mucosal immunity, where the outcome for the host is dependent on the specific challenge or cause of inflammation.

The host immune response to *C. rodentium* is composed of an early innate immune response, followed by the generation of CD4+ T cells and the eventual production of antibodies (23, 24). Although required for control and eventual clearance of the pathogen, these immune responses cause significant immunopathology (25, 26). In addition to the recruitment of various immune cell types, cytokines such as IFNγ induce crypt hyperplasia which is the rapid proliferation of intestinal epithelial cells (IEC), resulting in the loss of mature secretory and absorptive cell types in the colon (25, 27).

Here, we assessed the role of SP receptor signaling on the course of disease and the immune response elicited during infection with the noninvasive enteric bacterial pathogen *C. rodentium*. Using potent and selective TACR1 antagonists, we demonstrate significantly reduced *C. rodentium* burden, and infection-induced colonic pathology. Surprisingly, antagonism of the SP receptor significantly reduced selected aspects of the host response to this enteric bacterial pathogen, with decreased colonic T cell recruitment, and expression of critical cytokines 10 d postinfection (dpi). Analysis of the colon in these antagonist-treated and infected mice revealed significantly decreased expression of the adhesion molecule MAdCAM-1, which serves to recruit mucosal homing T cells to the intestine. In addition to reducing the recruitment of colonic T cells, significantly reduced IFNγ production and IFNγ+ CD4+ T cells were observed in the colon 10 dpi in TACR1 antagonist-treated mice compared to vehicle. These effects on T cells were further determined to not be due to changes in conventional dendritic cell (cDC) numbers or a reduced ability of these cells to present antigen, and T cells to respond in vivo to an orally administered antigen. With the recent demonstration that the SP receptor is required for T cell receptor-induced Ca^2+^ signaling (28), we investigated the effect of T cell–specific TACR1 deficiency during *C. rodentium* infection. Using a unique TACR1 T cell conditional KO mouse, we demonstrated that loss of T cell intrinsic SP receptor signaling does not reduce colonic T cell homing, but significantly attenuates T cell IFNγ production. Together, our data suggest that SP receptor signaling contributes to host defense through a variety of discrete cell types including T cells. Moreover, our data demonstrate that this receptor could be used to fine-tune immune responses during enteric bacterial infections to maintain host protection while reducing immunopathology.

## Results

### Inhibition of SP Receptor Signaling Reduces the Severity of *C. rodentium* Infection.

**Fig. 1.**
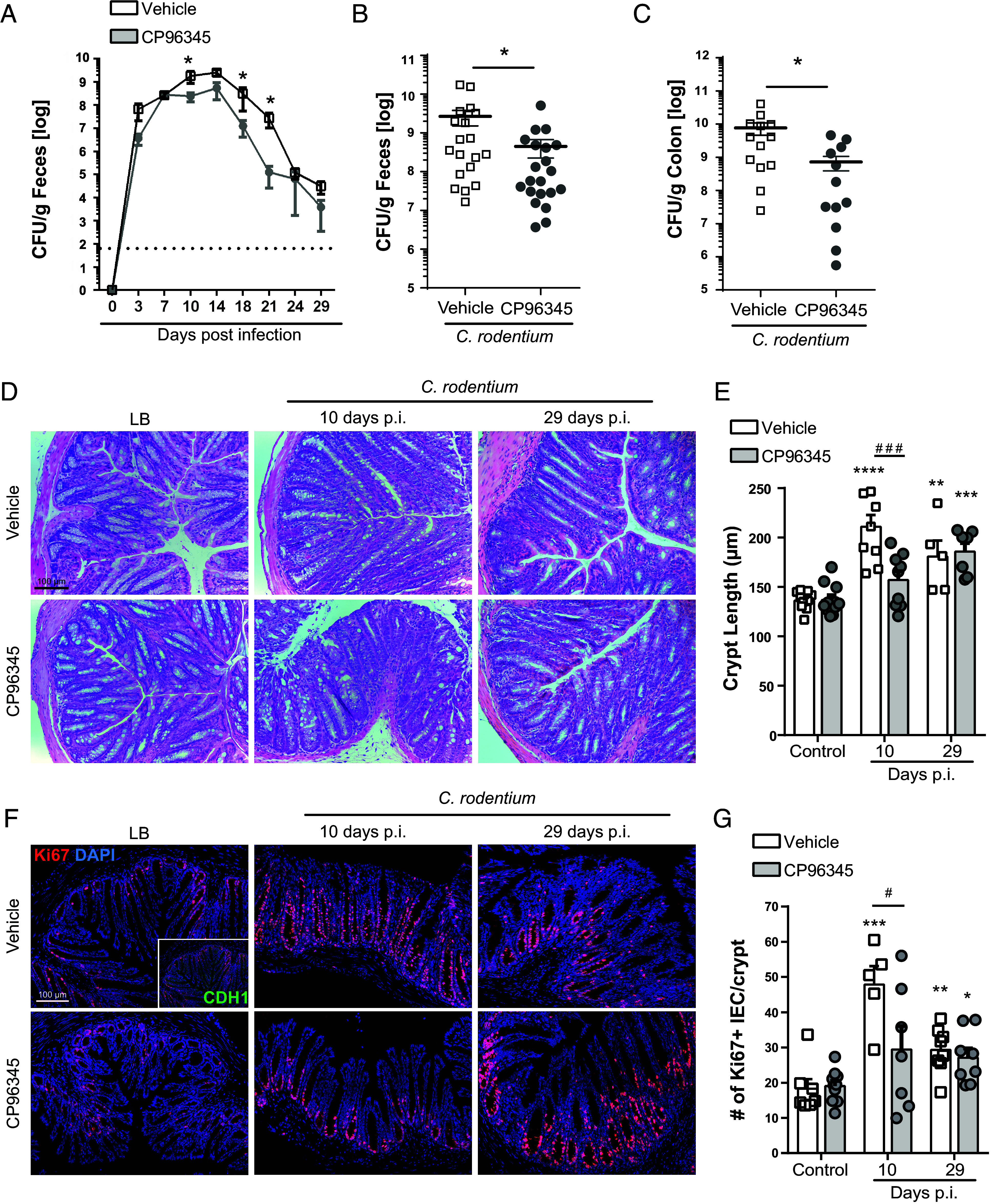
Antagonism of TACR1 signaling reduces *C. rodentium* burden and induced pathology. Administration of the highly selective TACR1 antagonist CP96345 (2.5 mg/kg, orogastric gavage, once daily) significantly reduces fecal *C. rodentium* over the course of infection (*A*). Reduced fecal (*B*) and colonic adherent (*C*) *C. rodentium* were observed in CP96345 compared to vehicle-treated mice at 10 dpi. Crypt length was measured on H&E-stained histology sections (*D*) and quantified (*E*) from vehicle or CP96345-treated uninfected or infected mice at 10 and 29 dpi. Immunofluorescent staining and confocal imaging were performed to identify (*F*) and enumerate (*G*) proliferating (DAPI+ Ki67+ CDH1+) IEC in uninfected or infected mice treated with vehicle or CP96345. Results are from individual mice, mean ± SEM. For (*A*–*C*) *P ≤ 0.05, **P ≤ 0.01, ***P ≤ 0.001. For (*D*–*G*), *P ≤ 0.05, **P ≤ 0.01, ***P ≤ 0.001 compared to uninfected controls of the same treatment and ^#^*P* ≤ 0.05, ^##^*P* ≤ 0.01, ^###^*P* ≤ 0.001 compared between treatment groups. Two-way ANOVA with Tukey’s post hoc test. (Scale bar, 100 µm.) Luria broth (LB), postinfection (p.i.), IEC.

To ascertain the role of SP receptor signaling in the host-response to enteric infection with *C. rodentium*, mice were treated with vehicle or the highly selective receptor antagonist CP96345 (2.5 mg/kg, daily orogastric gavage). Bacterial burden assessed in feces revealed that CP96345 treatment reduced bacterial burden throughout the infection, reaching statistical significance for 10-, 18-, and 21-dpi (Fig. 1*A*). In separate cohorts of mice, statistically significant reductions were again observed 10 dpi in fecal pellets (Fig. 1*B*), colonic tissues (Fig. 1*C*), and in the lumen of colonic tissue sections assessed by confocal microscopy (*SI Appendix*, Fig. S1 *A* and *B*) in CP96345 treated mice. As expected, vehicle-treated *C. rodentium–*infected mice had significantly increased colonic crypt hyperplasia compared to noninfected controls. In contrast, treatment with CP96345 reduced crypt hyperplasia 10 dpi (Fig. 1 *D* and *E*). These morphometric data were supported by significantly reduced numbers of proliferating (Ki67+) epithelial cell (CDH1+) cells in CP96345-treated *C. rodentium*–infected mice compared to vehicle 10 dpi (Fig. 1 *F* and *G*). While increased crypt length and epithelial cell proliferation were still observed at 29 dpi compared to uninfected controls, there was no difference between treatment groups in *C. rodentium*–infected mice 29 dpi. We confirmed that *C. rodentium* viability was not reduced by CP96345 at equivalent concentrations used in our in vivo studies (*SI Appendix*, Fig. S1*C*). Additionally, *C. rodentium* cultured in DMEM showed no defect in mRNA expression of the LEE transcriptional regulator *ler*, or the regulated genes *espA,* or *espB* in the presence of physiologically relevant concentrations of CP96345 when compared to vehicle controls (*SI Appendix*, Fig. S1 *D*–*F*). These data indicate that CP96345 does not impede the ability of *C. rodentium* to express these virulence genes. We further assessed colonic motility as a possible mechanism for reduced bacterial burden. We observed reduced distal colonic motor function in CP96345-treated mice (*SI Appendix*, Fig. S1 *G* and *H*) demonstrating that these reductions in bacterial burden are not simply due to increased colonic motor function. Highlighting that our observations are due to reduced TACR1 signaling and not a CP96345-specific effect, we observed reduced *C. rodentium* burden in mice treated with the TACR1 antagonist SR140333 (1 mg/kg) (29) delivered by intraperitoneal injection (*SI Appendix*, Fig. S1 *I* and *J*). *C. rodentium* is well known for increasing oxygen availability as a mechanism aiding bacterial colonization and pathogenesis. Given that we saw significant decreases in bacterial burden and pathology, we investigated whether oxygenation was a potential mechanism. We did not observe a difference in the staining of hypoxyprobe in CP96345-treated mice compared to respective controls (*SI Appendix*, Fig. S1*K*). Additionally, we investigated the impact the decrease in proliferation had on goblet cells (30) after *C. rodentium* infection. There was no difference in the number of goblet cells induced by TACR1 antagonist or by *C. rodentium* infection (*SI Appendix*, Fig. S2 *A* and *B*). Additionally, we assessed isolated distal IEC for the expression of *Atoh1* (*Math1*), the master regulator of secretory cell lineages including goblet cells. While *C. rodentium* infection significantly decreased *Atoh1* mRNA expression, there was no difference between treatment groups (*SI Appendix*, Fig. S2*C*). Highlighting the infection-induced changes that occurred in IEC, *C. rodentium* infection induced upregulation of MHCII mRNA on distal IEC which was significantly decreased in CP96345-treated mice 10 dpi (*SI Appendix*, Fig. S2*D*). Together these data demonstrate that TACR1 signaling is a critical element of host responses during enteric bacterial infection and blocking TACR1 signaling reduces bacterial burden and infection-induced pathology.

### Colonic T cell Recruitment Is Abrogated by Antagonism of the SP Receptor.

**Fig. 2.**
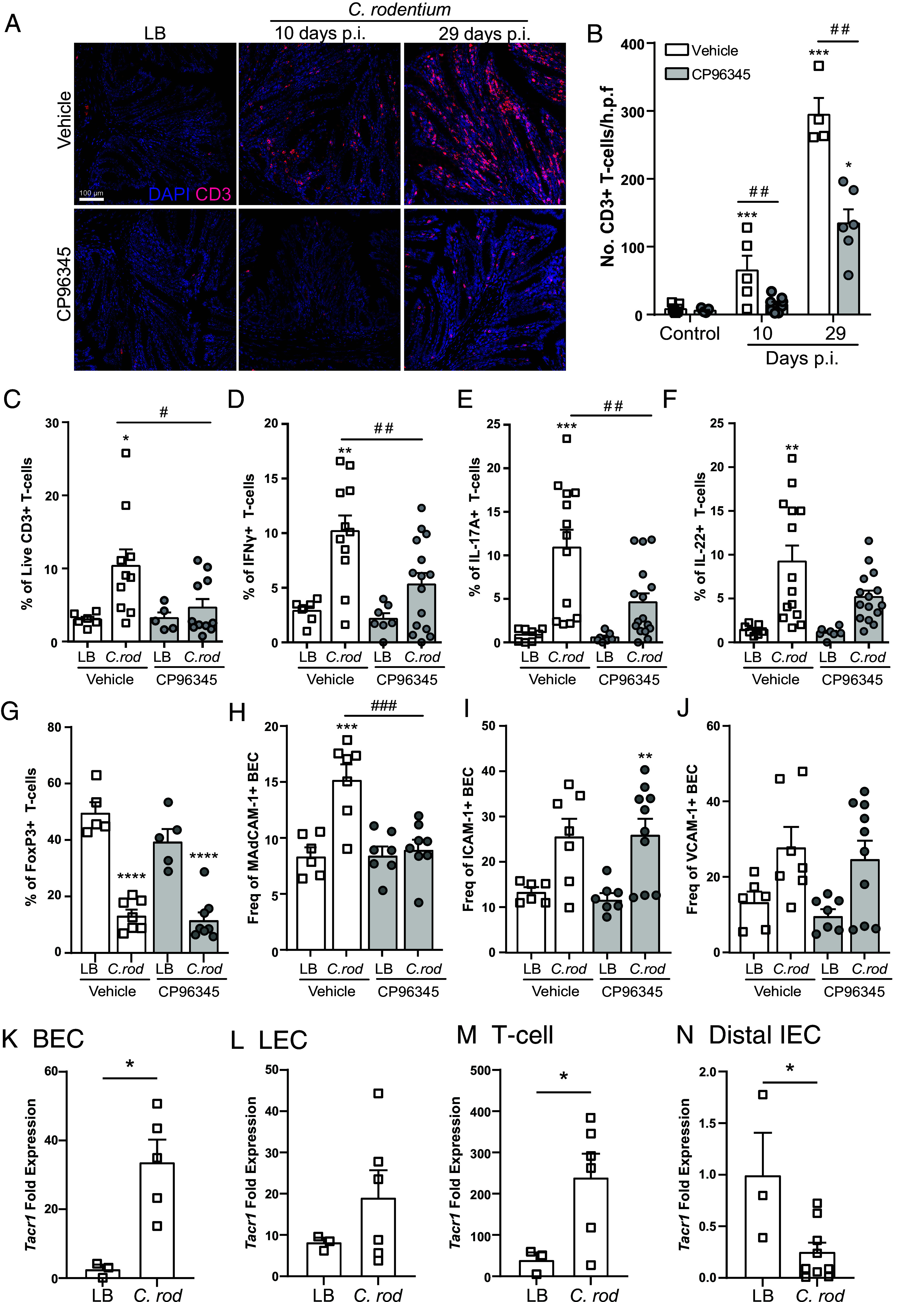
TACR1 signaling is required for recruitment of IFNγ and IL-17A producing T cells during enteric infection. Colonic recruitment of T cells (DAPI+ CD3+) in uninfected (LB) or *C. rodentium*–infected mice treated with vehicle or CP96345 was assessed in tissues sections obtained 10 and 29 dpi by immunofluorescence and confocal microscopy (*A*) and enumerated (*B*). LB or *C. rodentium–*infected mice treated with vehicle or CP96345 10 dpi were quantified via flow cytometry for T cell recruitment by frequency of live CD3+ T cells (*C*) as well as intracellular cytokine staining for CD4+ T cells that express IFNγ (*D*), IL-17A (*E*), IL-22 (*F*), and FoxP3 (*G*). In a separate cohort, colonic BEC (CD45−, CD31+ gp38−) from uninfected (LB) and *C. rodentium–*infected mice treated with vehicle or CP96345 10 dpi were analyzed for their expression of MAdCAM-1 (*H*), ICAM-1 (*I*), and VCAM-1 (*J*). *Tacr1* mRNA expression was assessed in sorted colonic BEC (*K*), lymphatic endothelial cells (LEC) (*L*), T cells (*M*), and distal IEC (*N*) in naïve (LB) or 10 dpi *C. rodentium* infected WT mice. Each data point represents an individual mouse. Mean ± SEM, *P ≤ 0.05, **P ≤ 0.01, ***P ≤ 0.001 compared to uninfected controls of the same treatment and ^#^*P* ≤ 0.05, ^##^*P* ≤ 0.01, ^###^*P* ≤ 0.001 compared between treatment groups. One-way ANOVA with Tukey’s post hoc test was used. (Scale bar, 100 µm.) Luria broth (LB), postinfection (p.i.), *C. rodentium* (*C. rod*).

Colonic crypt hyperplasia due to *C. rodentium* infection is well established to be driven by IFNγ-producing T cells (25, 27). With this in mind, we hypothesized that reduced colonic hyperplasia could be due to decreased T cell recruitment or production of these cytokines. Confocal microscopy revealed that, as expected, *C. rodentium* infection significantly increased the number of T cells (CD3+ DAPI+) in colonic tissue sections when compared to noninfected mice. However, the number of colonic T cells at 10 and 29 dpi with *C. rodentium* was significantly reduced in CP96345-treated mice compared to vehicle-treated controls (Fig. 2 *A* and *B*). These results were confirmed using flow cytometry demonstrating reduced numbers of CD3+ T cells (Live, singlet, CD45+, CD3+) in the lamina propria of infected mice treated with CP96345 compared to vehicle 10 dpi (Fig. 2*C*). Using ex vivo restimulation and intracellular cytokine staining, we identified that IFNγ and IL-17A, but not IL-22-producing CD4+ T cells were reduced in infected mice treated with CP96345 compared to vehicle-treated *C. rodentium*–infected controls (Fig. 2 *D*–*F*). Despite these changes in IFNγ and IL-17A, we found no effect of CP96345 on the prevalence of FoxP3+ T cells in the lamina propria (Fig. 2*G*) or in MLN (*SI Appendix*, Fig. S3*A*) across treatment groups. These data demonstrate that TACR1 antagonism reduces T cell recruitment and IFNγ and IL-17A cytokine production to reduce host-maladaptive immunopathology.

Reductions in bacterial burden early in the course of disease prior to when CD4+ T cells are recruited to the colon (Fig. 1*A*) led us to assess the ability of TACR1 antagonism to alter innate lymphoid cell (ILC) populations. Focusing on 3 dpi, a time point selected to occur before T cell responses, we found that CP96345 treatment did not affect the frequency of ILC1, ILC2, or NK cells in the colonic lamina propria regardless of *C. rodentium* infection (*SI Appendix*, Fig. S3 *B*–*D*). Further analysis demonstrated a significantly decreased frequency of natural cytotoxicity receptor (NCR)+ ILC3 and CD4− lymphoid tissue inducer (LTi) cells, with no significant decrease in NCR- ILC3 and CD4+ LTi cells in CP96345-treated compared to vehicle-treated mice (*SI Appendix*, Fig. S3 *E*–*H*). Thus, TACR1 receptor antagonism has marginal effects on specific ILC3 and LTi cell subpopulations.

Given the reduction of T cell recruitment into the lamina propria, we assessed the expression of cell adhesion molecules on the surface of BEC critical for T cell extravasation from the vasculature. By flow cytometry, we found a significant increase in frequency of ICAM-1+, VCAM-1+, and MAdCAM-1+ BEC (CD45−, gp38−, CD31+) in *C. rodentium*–infected mice 10 dpi compared to uninfected controls. BECs from infected mice receiving CP96345 upregulated ICAM-1 and VCAM-1 but had significantly reduced cell surface MAdCAM-1 expression compared to infected vehicle-treated controls (Fig. 2 *H*–*J*). Additionally, we found that sorted colonic BEC, LEC (CD45−, gp38+, CD31+), and T cells all express high levels of TACR1 mRNA relative to a known expressing ganglion. Specifically, BEC and T cells show significantly increased *Tacr1* expression 10 dpi with *C. rodentium* (Fig. 2 *K*–*M*). Conversely, distal IEC express *Tacr1* but significantly downregulate expression during *C. rodentium* infection (Fig. 2*N*). These data suggest that TACR1 antagonism reduced colonic T cell recruitment by reducing blood endothelial MAdCAM-1 upregulation in a cell-intrinsic manner.

### SP Receptor Signaling Attenuates Host Inflammation During *C. rodentium* infection.

**Fig. 3.**
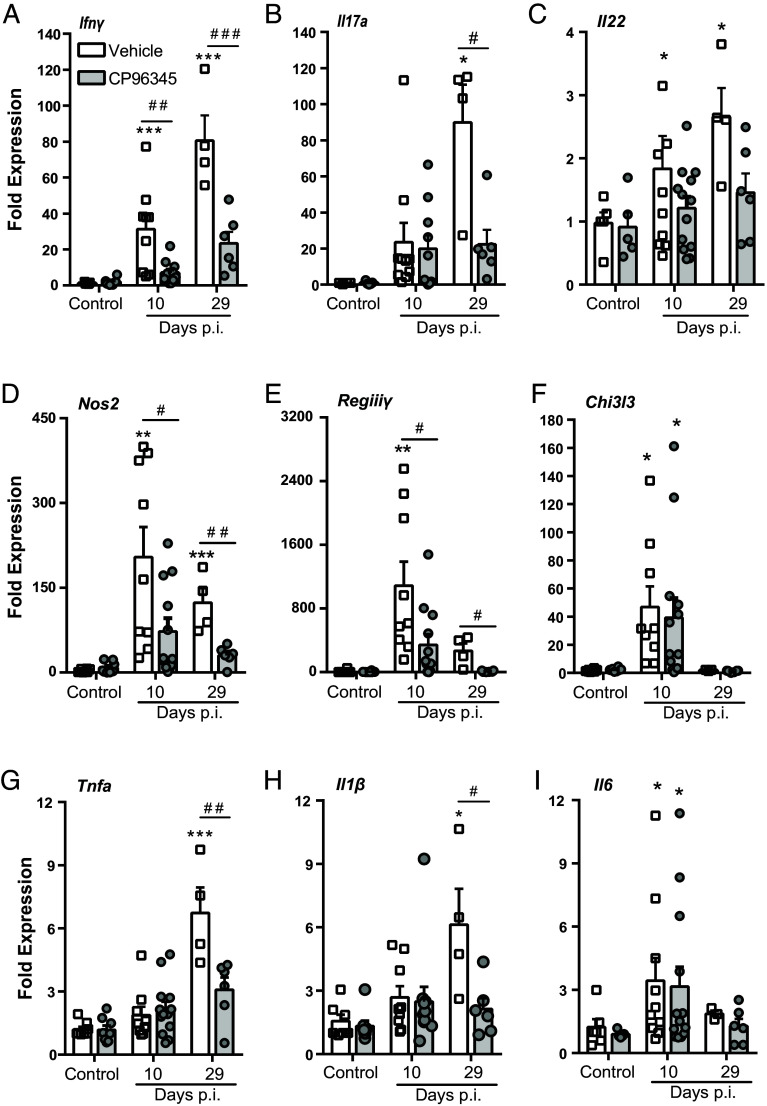
TACR1 blockade attenuates expression of specific proinflammatory genes in colonic tissues during *C. rodentium* infection. Colonic tissue from uninfected (LB) and infected mice treated with vehicle or CP96354 were assessed for expression of *Ifnγ* (*A*), *Il17a* (*B*), *Il22* (*C*) at 10 and 29 dpi. General inflammatory responses were assayed by measuring colonic expression of *Nos2* (*D*), *Regiiiy* (*E*), *Chi3l3* (*F*), *Tnfa* (*G*), *Il1β* (*H*), and *Il6* (*I*). Results are from individual mice, mean ± SEM, *P ≤ 0.05, **P ≤ 0.01, ***P ≤ 0.001 compared to uninfected controls of the same treatment and ^#^*P* ≤ 0.05, ^##^*P* ≤ 0.01, ^###^*P* ≤ 0.001 compared between treatment groups. Two-way ANOVA with Tukey’s post hoc test was used. Postinfection (p.i.).

With the reduced colonic T cell recruitment and MAdCAM-1 expression, we assessed whether CP96345 abrogated the expression of proinflammatory cytokines during *C. rodentium* infection. In keeping with our flow cytometry data, CP96345-treated *C. rodentium*–infected mice had significantly reduced *Ifnγ* mRNA compared to vehicle-treated infected mice 10 and 29 dpi (Fig. 3*A*). *Il17a* was also significantly decreased by CP96345 treatment at 29 dpi compared to vehicle-treated controls (Fig. 3*B*) while *Il22* was not affected by treatment at either timepoint (Fig. 3*C*). Confirming that these results were not specific to CP96345, administration of the TACR1 antagonist SR140333 reduced *Ifnγ* expression at 10 dpi compared to vehicle-treated *C. rodentium*–infected mice (*SI Appendix*, Fig. S4*A*), although SR140333 did not alter *Il17a* or *Il22* expression (*SI Appendix*, Fig. S4 *B* and *C*).

IFNγ is well appreciated to increase the expression of a variety of genes that are critical to host protection, including inducible nitric oxide synthase (iNOS, *Nos2*) and RegIIIγ (27, 31). Infection-induced increases in *Nos2* expression at 10 and 29 dpi were significantly reduced in CP96345-treated mice (Fig. 3*D*). *Regiiiγ* is a critical antimicrobial peptide often upregulated during *C. rodentium* infection in mice. We show similar upregulation of *Regiiiγ* induced by *C. rodentium* infection, but CP96345 treatment significantly reduced *Regiiiγ* expression at 10 and 29 dpi compared to vehicle (Fig. 3*E*). With TACR1 antagonism showing modulation of aforementioned genes related to macrophage function, we investigated *Chi3l3,* the alternatively activated macrophage marker gene, which was induced by *C. rodentium* infection but was not affected by treatment with CP96345 compared to vehicle controls at similar time points (Fig. 3*F*). In support of SP receptor regulation of specific gene expression profiles in response to enteric bacterial infection, *Tnfa* and *Il1β* expression induced by infection were significantly reduced by TACR1 antagonism 29 dpi compared to vehicle-treated and infected mice (Fig. 3 *G* and *H*). Expression of *Il6* induced by *C. rodentium* infection was not impacted by treatment with CP96345 (Fig. 3*I*). These results demonstrate TACR1 signaling during *C. rodentium* infection is a critical modulator of specific host-protective gene expression during inflammation, serving to shape the host response.

### Antagonism of SP Receptor Does Not Prevent Antigen-Specific T cell Proliferation In Vivo.

Here, we assessed whether reduced colonic T cell recruitment during TACR1 antagonist treatment was due to abrogated dendritic cell migration or function and antigen-specific T cell proliferation. No significant differences in frequency of live cDC (CD45+ CD3− B220− CD317− CD11c^hi^ MHCII^hi^) from the mesenteric lymph nodes (MLN) were identified 10 dpi in LB or *C. rodentium–*infected mice treated with vehicle or CP96345 (*SI Appendix*, Fig. S5*A*). There was also no significant difference in either protein expression of the costimulatory molecule CD86 by cDC (*SI Appendix*, Fig. S5*B*) or in the number of CD11b− or CD11b+ CD103+ migratory cDC (*SI Appendix*, Fig. S5*C*). To assess whether TACR1 antagonism reduced the function of DC to present antigen to naïve antigen-specific T cells, Cell Proliferation Dye eFluor450 labeled CD4+ OT-II T cells were adoptively transferred into WT mice and treated with vehicle or CP96354, then gavaged with ovalbumin or PBS (*SI Appendix*, Fig. S5*D*). TACR1 antagonism did not alter OT-II T cell proliferation within the MLN measured by proliferation and division index (*SI Appendix*, Fig. S5 *E* and *F*); however, there was a significant increase in the number of OT-II T cells in MLN in CP96345-treated mice compared to vehicle controls (*SI Appendix*, Fig. S5*G*). Together these data show that TACR1 antagonism neither alters the number and function of cDC, nor the ability to induce antigen-specific T cell proliferation but could reduce lymph node egress.

### T cell Intrinsic SP Receptor Signaling Enhances IFNγ Production.

**Fig. 4.**
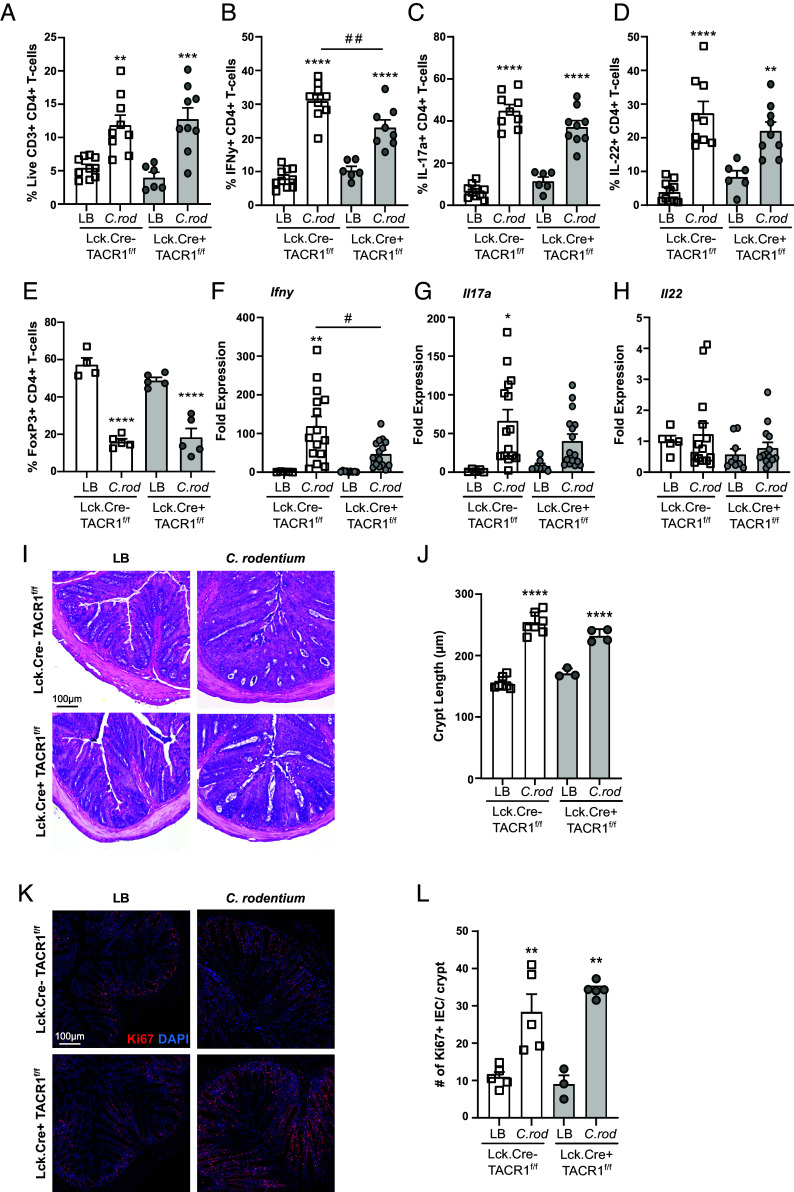
T cell intrinsic TACR1 signaling impacts IFNγ production during enteric infection. Colonic lamina propria lymphocytes were quantified in Lck.Cre+ TACR1^f/f^ mice and their Lck.Cre− TACR1^f/f^ littermates. Frequency of live CD3+ CD4+ T cells in the colonic lamina propria of *C. rodentium–*infected mice 10 dpi (*A*). Intracellular cytokine staining of IFNγ (*B*), IL-17A (*C*), IL-22 (*D*), and FoxP3 (*E*) producing CD4+ T cells 10 d post–*C. rodentium* infection. Colonic tissue from Lck.Cre+ TACR1^f/f^ mice and their Lck.Cre− TACR1^f/f^ littermates was assessed for mRNA expression of *Ifnγ* (*F*), *Il17a* (*G*), *Il22* (*H*) at 10 dpi. Colonic crypt length was measured in H&E-stained sections of LB or 10 dpi *C. rodentium–*infected Lck.Cre+ TACR1^f/f^ mice or Lck.Cre− TACR1^f/f^ littermates (*I* and *J*). DAPI+ Ki67+ CDH1+ IEC were stained (*K*) and enumerated (*L*) via immunofluorescent staining and confocal imaging in uninfected or infected Lck.Cre+ TACR1^f/f^ mice and their Lck.Cre− TACR1^f/f^ littermates 10 dpi. Results are from individual mice, mean ± SEM, *P ≤ 0.05, **P ≤ 0.01, ***P ≤ 0.001 compared to uninfected controls of the same treatment and ^#^*P* ≤ 0.05, ^##^*P* ≤ 0.01, ^###^*P* ≤ 0.001 compared between treatment groups. One-way ANOVA with Tukey’s post hoc test was used. (Scale bar, 100 µm.) Luria broth (LB), *C. rodentium* (*C. rod*).

To decipher the role of T cell intrinsic TACR1 signaling during *C. rodentium* infection, we used a conditional KO approach. TACR1 T cell conditional KO (Lck.Cre+ TACR1^f/f^) and WT (Lck.Cre− TACR1^f/f^) littermate controls were infected with *C. rodentium* and assessed 10 dpi. Flow cytometry conducted on colonic lamina propria lymphocytes revealed significant increases in T cell recruitment in both T cell cKO and WT mice compared to LB-treated controls with no difference between genotypes (Fig. 4*A*). In assessing cytokine production by intracellular cytokine staining and flow cytometry, the frequency of IFNγ+ T cells was significantly reduced in infected TACR1 T cell cKO compared to WT mice 10 dpi (Fig. 4*B*). Although a slight reduction in IL-17A+ T cells was also observed in infected TACR1 T cell cKO compared to WT mice 10 dpi, this was not statistically significant (Fig. 4*C*). As expected, *C. rodentium* infection increased IL-22+ colonic T cells; however, this was not different in WT compared to TACR1 T cell cKO mice (Fig. 4*D*). Regulatory FoxP3+ CD4+ T cells were also not different between WT or T cell TACR1 cKO mice in the lamina propria (Fig. 4*E*) and the MLN (*SI Appendix*, Fig. S6 *A* and *B*). These data were confirmed in qPCR conducted on colonic tissue samples, with infection-induced increases in *Ifnγ* expression in WT mice that were significantly reduced in TACR1 T cell cKO mice 10 dpi (Fig. 4*F*). Expression of *Il17a* and *Il22* showed no difference between *C. rodentium*–infected WT and TACR1 T cell cKO mice 10 dpi (Fig. 4 *G* and *H*). With the reduced IFNγ production by TACR1 T cell cKO compared to WT mice during infection, we assessed whether T cells from these mice were able to proliferate and differentiate to Th1 or Th17 T cells. Using in vitro polarizing conditions, we found no defect in TACR1 T cell cKO proliferation by EdU incorporation, or Th1 or Th17 differentiation indicated by production of IFNγ and IL-17A respectively (*SI Appendix*, Fig. S6 *C*–*F*). Interestingly, we found no significant difference in bacterial burden in these T cell cKO compared to WT mice (*SI Appendix*, Fig. S6 *G* and *H*). Infection with *C. rodentium* induced crypt hyperplasia, with no difference in crypt length in WT compared to TACR1 T cell cKO mice 10 dpi (Fig. 4 *I* and *J*). In keeping with these data, we also saw no difference in proliferating (Ki67+) IEC across genotypes (Fig. 4 *K* and *L*). Together, these data demonstrate that TACR1 cell-intrinsic signaling in T cells reduces select cytokine responses but is not sufficient to reduce bacterial burden and host pathology.

## Discussion

**Fig. 5.**
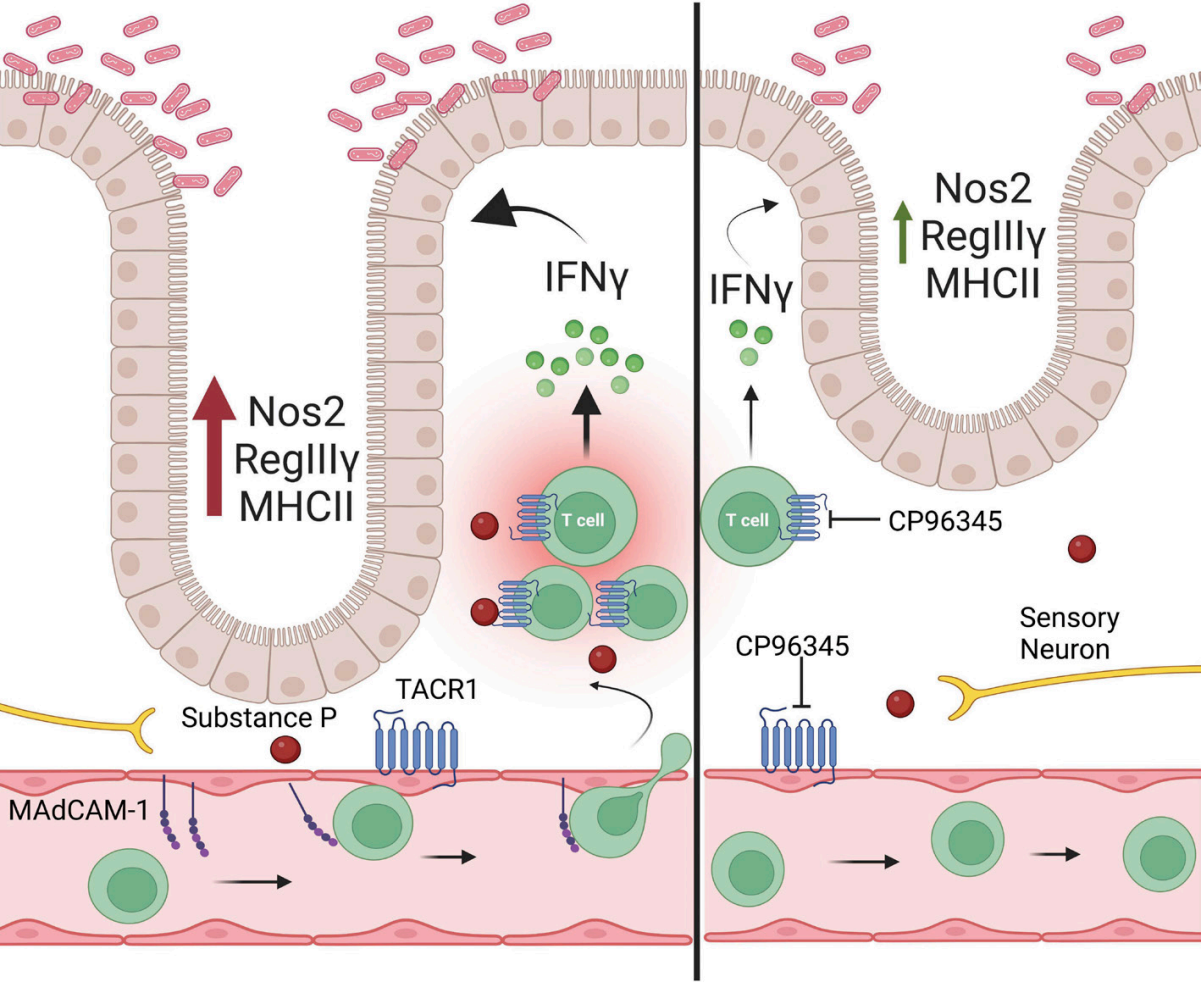
TACR1 signaling exacerbates inflammation, pathology, and bacterial burden. TACR1 signaling on BEC upregulates MAdCAM-1 which increases recruitment of T cells to the lamina propria during *C. rodentium* infection. TACR1 signaling on T cells enhances the expression of IFNγ which exacerbates crypt hyperplasia pathology and bacterial burden. Treatment with the TACR1 antagonist CP96345 reduces the recruitment of T cells by reducing the expression of MAdCAM-1 while also reducing IFNγ expression in T cells once in the lamina propria. These reductions lead to reduced crypt hyperplasia pathology and bacterial burden throughout the course of the infection.

Host-protective responses elicited by pathogens in the intestinal tract are tightly controlled and require a diverse array of cell types. Coordination of these responses can occur through many cell-intrinsic and cell–cell communication networks (32). Neurotransmitters released by the neurons in the ENS and extrinsic to the intestinal tract have become increasingly recognized to exert not only control over intestinal physiology but also immune function. We have previously demonstrated that ablation of sensory neurons expressing TRPV1 resulted in increased bacterial burden, and reduced T cell recruitment (10). This role of TRPV1 in sensory neurons was further demonstrated to elicit host protective recruitment of neutrophils during *C. rodentium* infection (11). These data suggested that there could be a complex array of signals that emanate from sensory neurons during enteric bacterial infection. SP has long been identified to function as a neurotransmitter critical in mediating neurogenic inflammation, intestinal physiology, and host-protective immune responses. The localized release of SP serves not only as a nociceptive neurotransmitter but also acts through the SP receptor on endothelial cells to increase vascular permeability, adhesion molecule expression, and vessel dilation (smooth muscle) (1). Despite these roles and our prior studies demonstrating the host protective role of TRPV1+ sensory nociceptors (10, 11), the specific function of SP during enteric bacterial infection was not well established. Our experiments indicate that SP receptor signaling modulates immune responses to *C. rodentium* infection that can be host maladaptive. Here, we demonstrate that SP receptor antagonism reduces bacterial burden and colonic crypt hyperplasia. This reduced bacterial burden was not simply due to bactericidal or bacteriostatic effects and could be replicated in experiments using a different SP receptor antagonist administered by i.p. injection. Additionally, we observed significantly decreased *Tnfα* and *Il1β* at the end of the infection, suggesting that SP receptor antagonism reduced bacterial burden and pathology leading to lasting effects in the host after the pathogen was cleared. These results are in keeping with recent findings where SP KO mice or, blockade of SP receptor signaling reduced intestinal inflammation induced by *Clostridium difficile* infection (33). Together these data demonstrate that SP receptor signaling is a critical determinant of bacterial burden during *C. rodentium* infection and suggest that TACR1 signaling can act in a host-deleterious manner (Fig. 5).

To discern how SP receptor antagonism may reduce *C. rodentium* bacterial burden and host pathology, we considered that treatment may limit the availability of host-derived factors required for maximal colonization by the pathogen (34–36). Proliferation of *C. rodentium* in the colonic lumen requires inflammation to increase oxygen availability in an otherwise hypoxic environment (37). However, there was no difference in colonic epithelial oxygenation in control or infected mice treated with SP receptor antagonist compared to vehicle controls. These data indicate that blocking SP receptor signaling reduces *C. rodentium* bacterial burden and inflammation, independent of increased colonic oxygenation.

Intestinal pathology during *C. rodentium* infection is not only due to the presence of this attaching and effacing pathogen but is also a consequence of the host’s immune response to the infection. Prior elegant studies have demonstrated that increased IEC proliferation that causes crypt hyperplasia is due to the recruitment of CD4+ T cells producing IFNγ and IL-22 (25, 26). This IFNγ-dependent immunopathology can however be fatal in the absence of macrophage and CD11b+ CD103− dendritic cells that produce IL-23 (38). Here, we demonstrate that SP receptor antagonism significantly reduced IEC proliferation (Ki67+ CDH1+), and consequently, infection-induced crypt hyperplasia at 10 dpi compared to infected vehicle-treated mice. This appeared to not be driven by IEC intrinsic TACR1 signaling as IEC expression of *Tacr1* was low and further downregulated during *C. rodentium* infection, suggesting that the crypt hyperplasia is driven by cell–cell interactions instead.

IFNγ signaling is well established to regulate the response to *C. rodentium* by acting on a variety of diverse cell types. As the primary interface between the host and bacterial pathogen, proteomic analysis on colonic IEC identified infection-induced increases in proteins regulated by IFNγ such as NOS2, RegIIIγ, and major histocompatibility complex II (MHCII). Although IFNγ deficiency did not increase bacterial burden at the height of the disease, IFNγ KO mice failed to increase MHCII on colonic epithelial cells and clear the infection. This requirement for MHCII expression by colonic IEC in protecting from *C. rodentium* infection was however discounted as IFNγ KO mice produced anti-*C. rodentium* antibodies and were protected from reinfection (27). Subsequent analysis confirmed that IFNγ signaling in IEC does not affect bacterial burden but instead is critical in driving intraepithelial lymphocytes to restrain infection-induced inflammation (39). Our data with SP receptor antagonists reducing colonic IFNγ and recruitment of IFNγ+ CD4+ T cells suggest that this is one facet of the immune response capable of reducing intestinal inflammation. Expression of MHCII on mid-distal IEC has also been suggested to present antigen to IL-22+ CD4+ T cells, sustaining cytokine release and aiding in the removal of susceptible IEC. Blockade of SP receptor signaling or conditional ablation of SP receptor on T cells reduced but did not ablate IFNγ production, suggesting that a fine balance of inflammation can be achieved, allowing for host protection without induction of immunopathology. Together these data indicate that host response to intestinal inflammation can be tuned through a variety of mechanisms including SP receptor signaling.

To understand the role of SP receptor signaling in T cell recruitment and given the known ability of SP to increase adhesion molecule expression that are critical to immune cell homing during intestinal inflammation (40, 41), we performed flow cytometry to assess the surface expression of these proteins on colonic BEC. In keeping with our prior results, *C. rodentium* infection increased adhesion molecule mRNA expression (10) and protein on the surface of colonic BEC (11). Compared to vehicle-treated *C. rodentium*–infected mice, MAdCAM-1 expression was significantly reduced on the endothelial cell surface of SP receptor antagonist-treated mice. Our data further suggest this may be driven by SP receptor signaling on BEC intrinsically as BEC express *Tacr1* mRNA which is further upregulated during *C. rodentium* infection. These results suggest that SP receptor signaling enhances colonic T cell recruitment by increasing MAdCAM-1 on the surface of endothelial cells. Although SP has been reported to increase adhesion molecule expression on BEC such as ICAM-1 and VCAM-1 (32, 40, 42), SP receptor antagonism did not reduce these cellular adhesion molecules induced by *C. rodentium* infection. It is critical to note that the SP-induced ICAM-1 and VCAM-1 expression were conducted on human dermal microvascular endothelial cells, or endothelial cell lines in vitro (40, 41). Mouse endothelial cell lines have also been shown not to increase ICAM-1 or VCAM-1 expression in response to SP (43). These differences could suggest that SP receptor signaling in intestinal BEC may elicit unique responses or that there are species-specific differences in SP receptor expression or signaling. Expression of MAdCAM-1 by BEC in the intestine has long been known as critical to the recruitment of mucosal homing T cells through interacting with T cells bearing α4β7 on their surface (44–47). MAdCAM-1 expression is increased in models of colitis, and colitis severity can be reduced through blockade or neutralization of α4β7. These findings therefore indicate that SP receptor signaling blockade can reduce infection-induced immunopathology by attenuating the expression of key adhesion molecules that recruit pathogenic T cells into the colonic lamina propria.

Using a SP receptor T cell cKO mouse, we further uncovered a unique role of T cell intrinsic signaling in *C. rodentium*–induced pathogenesis. Infected SP receptor T cell cKO mice had reduced colonic IFNγ expression and numbers of CD4+ IFNγ-producing T cells. Additionally, we found that lamina propria recruited T cells upregulate *Tacr1* mRNA during *C. rodentium* infection, suggesting that the enhanced IFNγ expression may be driven by local tissue signaling. However, recruitment of T cells to the infected colon is not SP receptor T cell intrinsic, as *C. rodentium* infection induced equivalent T cell recruitment in WT and cKO mice. These data are in agreement with previous publications demonstrating selective SP receptor antagonists significantly reduced antigen-specific IFNγ release upon T cell restimulation from *Schistosoma mansoni*–infected mice (48), and that SP enhances IFNγ production in T cells (49). Critically, we found that CD4+ T cells deficient in the SP receptor were still able to differentiate toward Th1 and Th17 T cells in vitro, indicating that SP can enhance but is not a requirement for T cell polarization. With these data and a prior report of SP receptor enhancing T cell receptor signaling (28), we assessed the effect of SP receptor antagonism on antigen-specific T cell proliferation in vivo. Our data demonstrate that antagonism of the SP receptor did not impact OT-II T cell proliferation in the MLN in response to orally administered ovalbumin, indicating that DC remains capable of antigen uptake, processing, homing to MLN, and presentation to T cells that remain responsive within these draining LN. Interestingly, we found a significant increase in the number of OT-II cells found in the MLN in mice treated with the SP receptor antagonist compared to vehicle control. This is in agreement with literature demonstrating that injection of SP into the lymph node increased lymph flow and trafficking of immune cells (50). These experiments demonstrate that the lack of recruited T cells, and consequently reduced immunopathology is not due to migratory DC deficits, or inability to activate T cells, but instead suggests defective T cell homing or emigration.

Together our data highlight that modulation of SP receptor signaling could be used to fine-tune immune responses in the intestine. Antagonism of the SP receptor allows for the development of host protection while attenuating immunopathology induced by enteric bacterial infection (Fig. 5). Modulation of immune responses through this signaling axis may prove amenable as our data adds to the preexisting literature indicating that SP enhances but is not required for immune function, allowing for antagonism without fear of inducing complete immunosuppression.

## Materials and Methods

### Mice.

Female and male 6- to 8-wk-old C57BL/6J, and Lck.Cre+ and Lck.Cre− TACR1^f/f^ mice on a C57BL/6J background were used in these studies. Mice were purchased from Jackson Laboratories (Bar Harbor, ME). Cryogenically frozen TACR1 embryos (EM:08399) were provided Consiglio Nazionale delle Ricerche as part of INFRAFRONTIER/EMMA (https://www.infrafrontier.eu/, PMID: 25414328). Mice were rederived at the Mouse Biology Program (UC Davis). Recovered mice were crossed to FLP mice on a C57BL/6J background to remove a Neo cassette and produce the conditional ready flanked by LoxP site line. These mice were further crossed to C57BL/6J mice, and progeny carrying the allele of interest were subsequently bred with Lck.Cre+ mice. All procedures and protocols were approved by the Institutional Animal Care and Use at UC Davis (#23469).

### *C. rodentium* Infection and Enumeration of Bacteria in Feces and Colon.

*C. rodentium*, strain DBS100, was kindly provided by Dr. Andreas Baumler. Bacteria were grown on MacConkey agar plates overnight at 37 °C, followed by an overnight culture in Luria broth (LB) at 37 °C without shaking. Mice were gavaged with LB or C. rodentium [10^8^ CFU (colony-forming units)].

Quantification of *C. rodentium* was performed with feces or 1 cm of distal colonic tissue placed into a preweighed tube and weighed to allow for determination of sample weight. Samples were homogenized using a stainless-steel bead (Qiagen, Germantown, MD) in a tissue lyser (Qiagen), followed by serial dilution and plating on MacConkey agar. Colonies were counted after 16 h of incubation at 37 °C, and CFU was calculated per gram of sample.

### TACR1 Antagonist Treatment.

Cohorts of mice received vehicle (DMSO diluted in PBS) or the selective SP receptor antagonist CP96345 2.5 mg/kg by orogastric gavage (Tocris, Minneapolis, MN) from the day of infection until the end of the experiment (51, 52). Additional cohorts of mice received vehicle or the selective SP receptor antagonist SR140333 (Tocris, Minneapolis, MN) by intraperitoneal injection 1 mg/kg every 2 d for the duration of the experiment (53).

### Gastrointestinal Motor Function.

Distal colonic motor function was measured in uninfected vehicle- and antagonist-treated mice after the second dose of the drug, by assaying the number of fecal pellets, in a 20-min period. Additionally, pellet weight was measured without dehydration.

### RNA Isolation and qPCR.

Tissues removed during necropsy were placed immediately into TRIzol and frozen until RNA extraction was performed. Samples were thawed, and a stainless-steel bead added to allow for tissue disruption and homogenization in a tissue lyser (Qiagen). RNA extraction was completed as instructed by the manufacturer (Invitrogen, Carlsbad, CA), with quantification by NanoDrop. Synthesis of cDNA was performed using an iScript kit with 1 μg of template RNA, following manufacture instructions (Biorad, CA). This cDNA was used to evaluate target gene expression by RT-qPCR with SYBR green incorporation using primer pairs from Primerbank (*SI Appendix*, Table S1).

### Histology.

Tissues dissected at necropsy were fixed in 10% normal buffered formalin, processed, and embedded in Paraffin according to standard protocols. Colonic tissues (1 cm in length) were cut into 3 to 5 pieces and embedded on end diagonally to allow for cross sections to be obtained. Paraffin embedded samples were first trimmed of 50 μm to achieve a flat surface and then sectioned by microtome to produce 6 μm thick sections on slides for histopathology or confocal microscopy. Sections for histopathology were deparaffinized, rehydrated, and stained with hematoxylin and eosin following standard protocols. Colonic epithelial cell hyperplasia was assessed by bright field microscopy to allow for measurements of crypt length in at least 15 and a maximum of 30 well-orientated crypts per sample with FIJI (Fiji is just ImageJ, NIH). Well-orientated crypts were defined as the base of crypt is visible and can be followed uninterrupted to the top of the crypt table. Two to three sections were analyzed at random, and an average was produced per animal.

### Confocal Microscopy.

Distal colonic tissue samples for confocal microscopy were prepared as previously described (11). In brief, after slides with 6 μm thick sections were deparaffinized and rehydrated, antigen retrieval was performed in citrate buffer (10 mM, pH 6.0, 30 min., 95 °C). After blocking in 5% BSA and normal donkey or goat serum (1 h RT), samples were incubated in primary antibody overnight (16 h, 4 °C). Primary and secondary antibodies used are detailed in *SI Appendix*, Table S2. Slides were washed extensively (3 × 5 min) in TBS-tween20 and incubated in appropriately labeled secondary antibodies (Invitrogen, Carlsbad, CA) for 1 h at RT, washed, counterstained with DAPI (1:5,000 TBS-tritonX100 0.1% v/v), washed extensively, and mounted in Prolong gold (Invitrogen, Carlsbad, CA). Staining using anti-mouse CDH1 (E-cadherin) was revealed using a mouse-on-mouse kit according to the manufacturer’s instructions (Vector laboratories, Burlingame, CA). Slides were imaged on a Leica SP8 STED 3× confocal microscope with a 40 × 1.3 NA objective, with optimal excitation provided by a white-light laser, or a 405 nm laser for DAPI, and appropriate emission collected for each fluorophore. Areas larger than the field-of-view of the objective were acquired using a tiling approach, whereby adjacent images were acquired with a 10% overlap. Files were processed by Imaris Stitcher (Oxford Instruments, UK) to recreate a composite image of the sample. Confocal data analysis was performed by importing Leica image format files into Imaris Stitcher (v9.0, Oxford Instruments) allowing for fusion of overlapping fields-of-view together. Two to three sections were chosen at random and averaged to represent each animal. Expression of Ki67 in IEC was determined by creating a mask in Imaris (Oxford Instruments) of IEC defined by DAPI+ CDH1+ cells. This masked region was then interrogated for the number of Ki67+ cells. Total Ki67+ (proliferating) cells (54) were divided by the number of crypts defined within the region. CD3+ DAPI+ T cells were enumerated by manual counting.

### Colonic Hypoxyprobe Imaging.

To determine the extent of colonic oxygenation, Hypoxyprobe Kit (Cat# HP1-XXX, Hypoxyprobe Inc., Burlington, MA) was used. The manufacturer’s protocol was followed. Briefly, WT mice were infected with *C. rodentium* or LB control and received CP96345 or vehicle as described above. After 10 dpi, mice received 2 mg of pimonidazole intraperitoneally 30 min before euthanasia, followed by removal and fixation of the colon. Tissues were processed as described above, and Paraffin-embedded sections were stained with mouse anti-pimonidazole (1:50 v/v dilution) followed by detection with streptavidin Alexa Fluor 647, and DAPI to reveal nuclei. Immunofluorescence was imaged as described above and files processed by Imaris (Oxford Instruments).

### Goblet Cell Enumeration.

Colon tissue sections from above were stained using Period Acid-Schiff stain protocol. In brief, paraffin-embedded sections were rehydrated from Histo-Clear rinses followed by decreasing concentrations of IPA and finally H_2_O. Slides were then incubated in 1 g/dL Periodic Acid for 5 min, then washed in H_2_O for 1 min three times. Next, sections were incubated in Schiff reagent for 15 min, then washed in H_2_O extensively before being stained by hematoxylin. Slides were imaged on the Nikon Eclipse E400 microscope using Spinview software (London, UK). Goblet cells stained dark purple in well-oriented crypts as described above were manually counted in FIJI (ImageJ) in two representative images. The number of goblet cells’ total was divided by the number of well-oriented crypts per image, and then the two representatives of one animal were averaged.

### Viability of *C. rodentium*.

The viability of *C. rodentium* in the presence of vehicle (DMSO) or CP96345 was assessed by the addition of either compound to the bacterial culture. A single colony of *C. rodentium* was cultured overnight in LB at 37 °C statically. The bacterial culture was diluted 1:10 in fresh LB with increasing concentrations of CP96345 or DMSO and incubated for 2.5 h at 37 °C statically. Culture samples were serially diluted and plated on MacConkey agar plates for overnight incubation before CFU was enumerated.

### Assessment of *C. rodentium* LEE Pathogenicity Gene Expression.

A single colony of *C. rodentium* is cultured in LB overnight at 37 °C statically. Bacteria are then diluted 1:20 into 10 mL DMEM (Cat# 11995-065 ThermoFisher, Waltham, MA) or LB with increasing concentrations of CP96345 or DMSO and cultured at 37 °C for 3 h 200 RPM. Bacteria are pelleted by centrifugation and resuspended in TRIzol reagent. RNA is extracted as described in manufacturer protocol and cDNA generated with iScript reverse transcriptase as described above. Primers for *espA*, *espB*, and *ler* were normalized to the housekeeping gene *rrsA* (*SI Appendix*, Table S1). Expression was compared to the control grown in DMEM with equivalent concentrations of DMSO, whereby a Fold Expression of 1 means there is no difference between CP96345 or its DMSO control.

### T cell Enrichment.

Inguinal, MLN and spleen were sterilely excised from nontreated Lck.Cre+ TACR1^f/f^ and Lck.Cre− TACR1^f/f^ mice and placed on a 100 μm filter and dissociated using the plunger of a syringe followed by several washing steps with stain buffer (1X PBS + 2% FBS). Single-cell suspensions were treated with Ack lysis solution (5 min RT), before being washed. Cells were then incubated with Fc block (anti-CD16/32, 10 µg/mL, Tonbo Biosciences, San Diego, CA) for 15 min on ice. Antibody cocktail was added to surface stain cells for 30 min on ice, followed by extensive washing in stain buffer. The following biotinylated antibodies were added to this cocktail at 1:50 dilution: anti-CD161 (clone# PK136, Ref# 30-5941), anti-CD11c (clone# N418, Ref# 30-0114), anti-Ly6G (Clone# RB6-8C5, Ref# 30-5931), anti-TER119 (Clone# TER-119, Ref# 30-5921), anti-CD11b (Clone# M1/70, Ref# 30-0112), and anti-B220 (Clone# RA3-6B2, Ref# 30-0452) from Tonbo Biosciences. Cells were then washed and incubated in magnetic streptavidin beads (Cat# 557812, BD Biosciences, Franklin Lakes, NJ) according to the manufacturer’s protocol. Cells were placed in BD IMAG Cell Separation Magnet (Cat# 552311 BD Biosciences, Franklin Lakes, NJ) for 8 min and the negative fraction was taken and placed into a fresh tube on the IMAG. This process was repeated for two additional magnetic enrichment steps to reach a T cell purity of >90% confirmed by flow cytometry.

### In Vitro T cell Differentiation and ELISA.

Negatively enriched CD4+ T cells were plated on untreated round bottom 96-well plates at 25,000 cells/well. Wells were pretreated with 2 µg/mL anti-CD3 (Cat# 40-0032 Tonbo Biosciences, San Diego, CA) in PBS for 2 h 37 °C 5% CO_2_. Cells were cultured with or without 0.5 µg/mL anti-CD28 (Cat# 40-0281 Tonbo Biosciences, San Diego, CA) and in Th1 or Th17 skewing culture conditions. Th1 consisted of 1 µg/mL anti-murine IL-4 (Cat# 16-7041 Invitrogen, Carlsbad, CA), 5 ng/mL murine IL-2 (Cat# 212-12 Peprotech Cranbury, NJ), and 10 ng/mL murine IL-12p40 (Cat# 210-12 Peprotech Cranbury, NJ). Th17 consisted of 1 µg/mL anti-murine IFNγ (Cat# 16-7312 Invitrogen, Carlsbad, CA), anti-murine IL-4 (Cat# 16-7041 Invitrogen Carlsbad, CA), 1 µg/mL anti-murine IL-2 (Cat# 14-7022 Invitrogen, Carlsbad, CA), 20 ng/mL murine IL-6 (Cat# 216-16 Peprotech, Cranbury, NJ), and 1 ng/mL murine TGF-β1 (Cat# 7666-MB R&D Systems, Minneapolis, MN). Cells were incubated at 37 °C 5% CO_2_ for 96 h, and then stimulated with 1× Cell Stimulation Cocktail (phorbol 12-myristate 13-acetate) (eBioscience, San Diego, CA) for 4 h. Supernatant was frozen at −20 °C until loaded into an ELISA. Untreated 96 flat bottom plates were coated overnight at 4 °C with capture antibody for IFNγ or IL-17A from Invitrogen kits (Cat# 88-7314-22 and Cat# 88-7371-88 respectively from Invitrogen, Carlsbad, CA) and manufacturer protocols were followed. Plates were read on a BioTek Synergy HTX Multi-mode Plate Reader using Gen5 application by the 450 nm and 570 nm wavelengths. 450 nm wavelength was subtracted from the 570 nm wavelength and the standard curve was determined using a 4-parameter logistic fit.

### In Vitro T cell Proliferation Analysis.

Splenic, mesenteric, and inguinal lymph node negatively selected CD4+ T cells from Lck.Cre+ TACR1^f/f^ mice and Lck.Cre− TACR1^f/f^ littermates were cultured in 96-well round bottom plates coated with 2 µg/mL anti-CD3 and with or without 0.5 µg/mL anti-CD28 as described above. Cells were cultured for 48 or 72 h before adding 10 µM EdU into culture media. Detection of incorporated EdU was performed according to the manufacturer’s instructions (Click-IT EdU AF647 Cat# C10340 Invitrogen, Carlsbad, CA) by flow cytometry.

### Isolation of Cells from the Colon.

To reduce colonic tissue to a single-cell suspension, lamina propria dissociation kit (Miltenyi Biotec, Gaithersburg, MD) was used with a gentleMACS tissue dissociator. The manufacturer’s protocol was followed. In brief, colons were excised and opened longitudinally, then cut into 1 cm long segments. Epithelial cells removed by gentle agitation (200 RPM) in HBSS supplemented with 10 mM HEPES, 5 mM EDTA, and 5% FBS and saved for RNA extraction in TRIzol reagent. Tissue was digested using MACS digestion enzyme mix as described in manufacturer’s protocol and reduced to a single-cell suspension by the gentleMACS device. Cell suspension was passed through a 100 µm strainer, washed extensively, and subjected to staining.

### Flow Cytometry.

A standard flow cytometry staining protocol was followed. Cells were counted manually by hemocytometer with trypan blue exclusion and single-cell suspensions incubated with Fc block (anti-CD16/32, 10 µg/mL, Tonbo Biosciences, San Diego, CA) for 25 min on ice before incubation with antibody cocktail (*SI Appendix*, Table S3) for 30 min on ice. Viability was determined using live/dead aqua according to the manufacturer’s instructions (ThermoFisher, Waltham, MA). Cells were fixed using BD Cytofix (BD Biosciences, Franklin Lakes, NJ) for 25 min on ice. All flow cytometry data were acquired on an LSRII (BD Biosciences, Franklin Lakes, NJ) using DIVA software, with analysis using FlowJo (Becton Dickinson, Eugene, OR).

### Intracellular Staining.

Prior to surface staining, colonic single-cell suspension was incubated in RPMI media with 10% FBS, 1% Penicillin/ Streptomycin, 2 mM L-glutamine, and BD GolgiPlug (1:500, Cat# 555029, BD Biosciences, Franklin Lakes, NJ) for 4 h at 37 °C 5% CO_2_ and stimulated with 1× Cell Stimulation Cocktail (phorbol 12-myristate 13-acetate) (eBioscience, San Diego, CA). Following surface staining, cells were fixed and permeabilized using a BD Cytofix/Cytoperm Fixation/Permeabilization Solution kit (Cat# 554714 BD Biosciences, Franklin Lakes, NJ) followed by intracellular staining with anti-IFNγ, anti-IL-17a, and anti-IL-22 (*SI Appendix*, Table S3) in 1× BD Perm/ Wash Buffer (Cat# 554723 BD Biosciences, Franklin Lakes, NJ) for 1 h. For FoxP3 staining, cells were fixed and permeabilized using the FoxP3 Transcription Factor Staining Kit (Cat# 00-5523 Thermofisher, Waltham, MA) for 1 h at 4 °C and then stained with anti-FoxP3 for 1 h at room temperature. Cells were then extensively washed and analyzed on an LSRII (BD Biosciences, Franklin Lakes, NJ) using DIVA software.

### TACR1 mRNA Expression Determination.

Naïve control (LB) and 10 dpi *C. rodentium* infected WT mice were euthanized, and colons excised. A single-cell suspension was achieved as described above and stained with anti-CD3, anti-CD45, anti-gp38, anti-CD31, and Live/Dead Fixable Aqua. BEC (CD45−, CD31+, gp38−), LEC (CD45−, CD31+, gp38+), and T cells (CD45+ CD3+) were sorted into separate tubes using an Astrios Cell Sorter (Beckman Coulter, Brea, CA). Distal IEC were removed as described above, washed, and stored in TRIzol. RNA was extracted and processed for qPCR analysis using the Takara CellAmp Direct TB Green RT-qPCR Kit (Cat# 3735A) according to the manufacturer’s protocol.

### Antigen-Specific T cell Proliferation In Vivo.

C57BL/6J mice were pretreated with 2.5 mg/kg CP96345 or vehicle (DMSO: PBS) for 3 d followed by another 4 d of treatment daily. After 3 d of pretreatment, negatively purified splenic and lymph node CD4+ T cells from OT-II mice were adoptively transferred to these WT pretreated mice via retro-orbital injection of 10^7^ cells. OT-II cells were negatively purified as described in T cell enrichment but underwent an additional incubation with Cell Proliferation dye eFluor450 (Cat# 65-0842-85 eBioscience, San Diego, CA) following the manufacturer’s protocol. One day later, 100 mg of ovalbumin in 200 μL PBS was administered intragastrically (i.g.). Three days later, MLN were excised, reduced to a single-cell suspension by mechanical force, and stained for flow cytometry. CD45+, CD3+ CD4+ Vα2+, Vβ5+ cells were quantified for their proliferation cycles using FlowJo’s cell cycle tool and proliferation and division index were calculated.

### Statistics.

Statistical analysis of all data was performed using Prism 10.0 (GraphPad, La Jolla, CA) with a Student’s *t* test or one-way or two-way ANOVA followed by post hoc analysis with Tukey’s multiple comparison test. Individual data points are presented as mean ± SEM.

## Supplementary Material

Appendix 01 (PDF)

## Data Availability

All study data are included in the article and/or *SI Appendix*.
