# Supplementary material for: Substance P receptor signaling contributes to host maladaptive responses during enteric bacterial infection
Source: Proc Natl Acad Sci U S A. 2025 Feb 12;122(7):e2415287122. doi: 10.1073/pnas.2415287122 (PMC11848390; doi:10.1073/pnas.2415287122)
Supplement: Supplementary file 1 — Appendix 01 (PDF) [file pnas.2415287122.sapp.pdf]

Figure S1

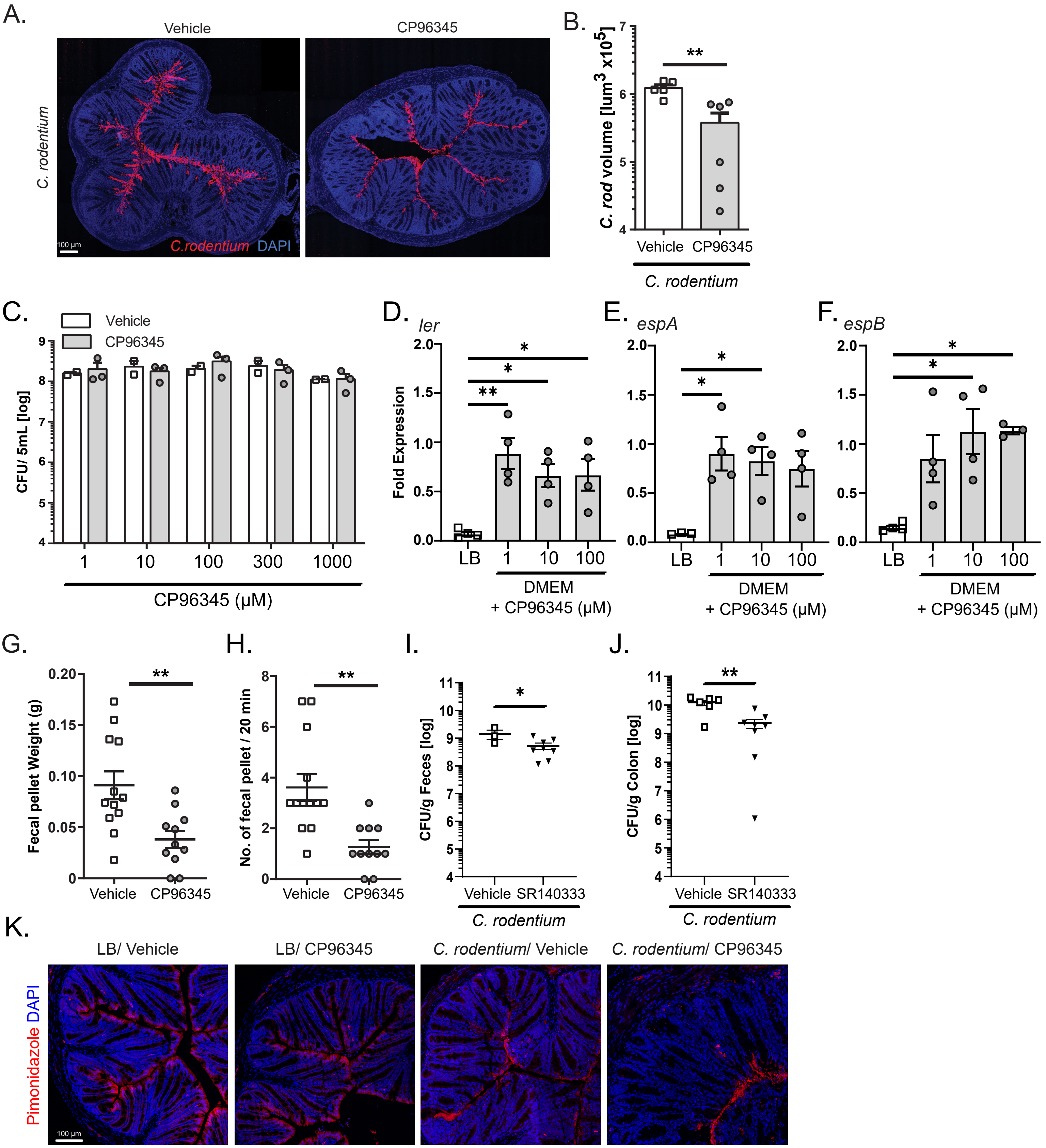

A.
Vehicle
CP96345
C. rodentium
100 µm
C.rodentium DAPI
B.
C. rod volume [µm^3 x10^5]
**
Vehicle
CP96345
C. rodentium
C.
Vehicle
CP96345
CFU/ 5mL [log]
1
10
100
300
1000
CP96345 (µM)
D.
ler
Fold Expression
LB
DMEM
+ CP96345 (µM)
E.
espA
F.
espB
G.
Fecal pellet Weight (g)
H.
No. of fecal pellet / 20 min
I.
CFU/g Feces [log]
Vehicle
SR140333
J.
CFU/g Colon [log]
K.
LB/ Vehicle
LB/ CP96345
C. rodentium/ Vehicle
C. rodentium/ CP96345
Pimonidazole DAPI

Figure S1. CP96345 reduces *C. rodentium* burden and GI motility without affecting bacterial viability or IEC oxygen availability. Immunofluorescence staining of colonic tissue sections with anti-*Citrobacter rodentium* specific antibodies were performed and images acquired with confocal microscopy **(A)** and the volume of stained bacteria quantified **(B)**. *C. rodentium* viability was quantified in the presence of CP96345 or vehicle (DMSO) in liquid culture by plating serial dilutions and enumerating CFU **(C)**. *C. rodentium* was cultured in LB or DMEM with increasing concentrations of CP96345 to assess expression of *ler* **(D)**, *espA* **(E)**, and *espB* **(F)** mRNA was normalized to its equivalent percentage of DMSO control. Distal colonic motor function was measured in mice treated with vehicle or CP96345 by fecal pellet weight **(G)** and number of fecal pellets excreted in 20 minutes **(H)**. Fecal pellets **(I)** and colonic tissue **(J)** were quantified for *C. rodentium* burden 10 dpi in mice treated with vehicle or another TACR1 antagonist SR140333 intraperitoneally every two days. The degree of infection-induced oxygenation in the colonic tissues was determined by injection of Hypoxyprobe into uninfected and infected, vehicle or CP96345-treated mice 10 dpi. This probe was detected with immunofluorescence to detect the Hypoxyprobe adduct (pimonidazole), and images acquired by confocal microscopy **(K)**. Results are from individual mice or individual bacterial colonies, mean ± SEM, * = $P \leq 0.05$, ** = $P \leq 0.01$, *** = $P \leq 0.001$. One-way ANOVA with Tukey's post-hoc test was used. Luria broth (LB), *C. rodentium* (*C. rod*).

Figure S2

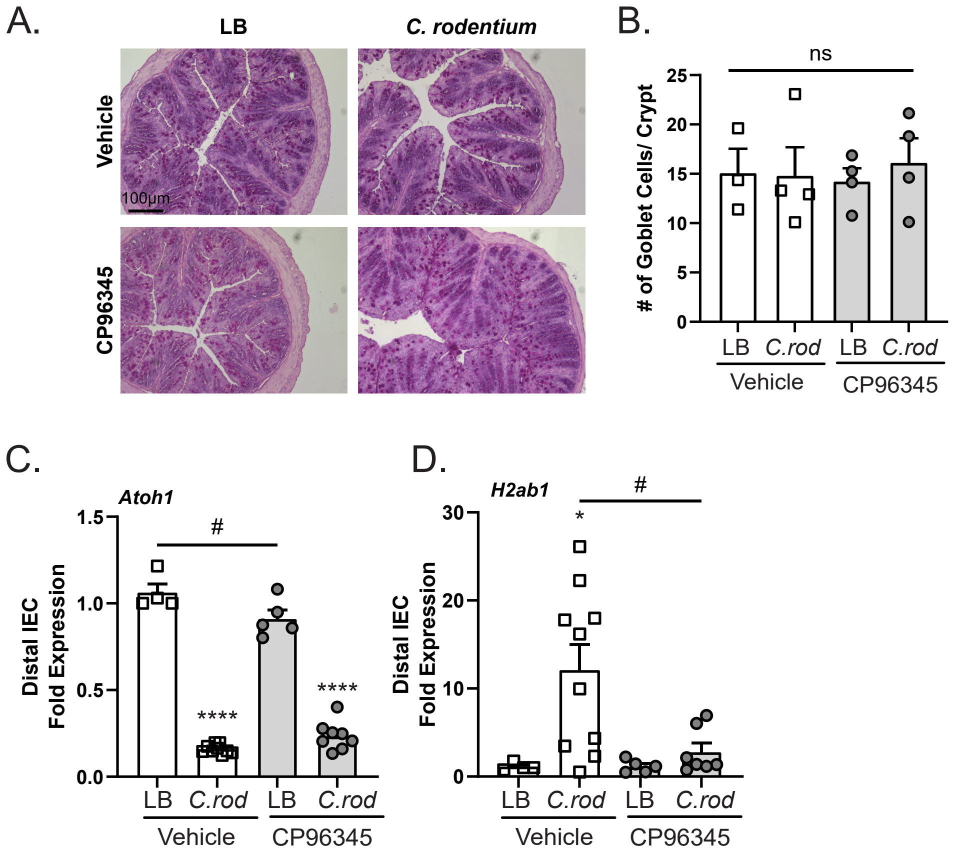
A.
LB
C. rodentium
Vehicle
CP96345
100μm
B.
ns
# of Goblet Cells/ Crypt
LB
C.rod
LB
C.rod
Vehicle
CP96345
C.
Atoh1
Distal IEC Fold Expression
#
****
****
LB
C.rod
LB
C.rod
Vehicle
CP96345
D.
H2ab1
Distal IEC Fold Expression
#
*
LB
C.rod
LB
C.rod
Vehicle
CP96345

Figure S2. TACR1 inhibition does not impact goblet cells. Colonic sections from mice treated with CP96345 or vehicle and infected with *C. rodentium* or LB 10 dpi were assessed for goblet cell numbers via PAS staining **(A)** and enumerated **(B)**. Isolated distal colonic epithelial cells from the tissue of 10 dpi *C. rodentium* or LB mice that were given CP963345 or vehicle and assessed for mRNA expression of *Atoh1* **(C)** and *H2ab1* **(D)**. Results are from individual mice, mean ± SEM, * = P ≤0.05, ** = P ≤0.01, *** = P ≤0.001 compared to uninfected controls of the same treatment and # = P ≤0.05, ## = P ≤0.01, ### = P ≤0.001 compared between treatment groups. One-way ANOVA with Tukey's post-hoc test was used. Luria broth (LB), *C. rodentium* (*C. rod*). Scale bar = 100μm.

Figure S3

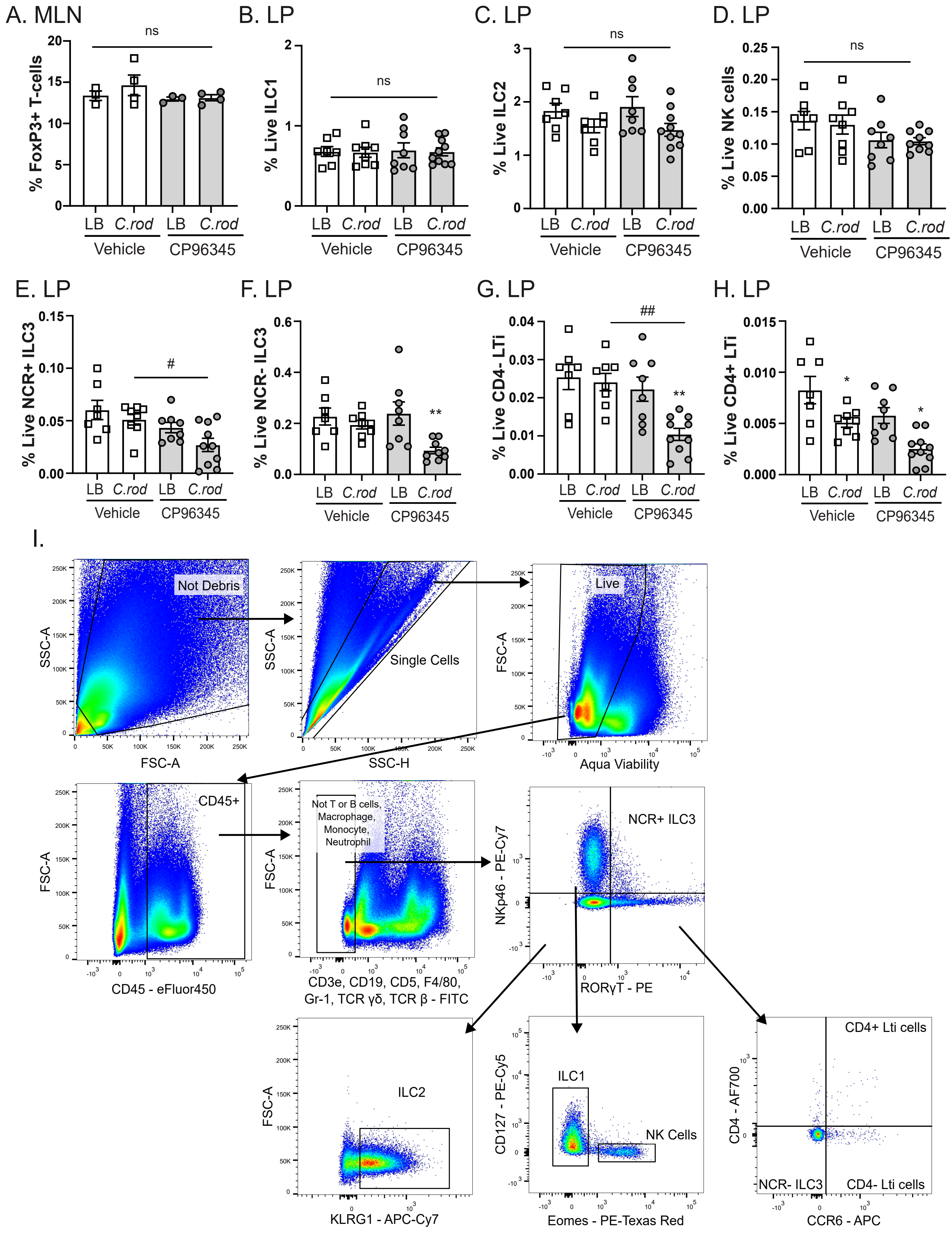
A. MLN
% FoxP3+ T-cells
ns
B. LP
% Live ILC1
ns
C. LP
% Live ILC2
ns
D. LP
% Live NK cells
ns
E. LP
% Live NCR+ ILC3
#
F. LP
% Live NCR- ILC3
**
G. LP
% Live CD4- LTi
##
**
H. LP
% Live CD4+ LTi
*
*
LB C.rod LB C.rod
Vehicle CP96345
I.
Not Debris
SSC-A
FSC-A
Single Cells
SSC-H
Live
Aqua Viability
CD45+
CD45 - eFluor450
Not T or B cells, Macrophage, Monocyte, Neutrophil
CD3e, CD19, CD5, F4/80, Gr-1, TCR γδ, TCR β - FITC
NCR+ ILC3
NKp46 - PE-Cy7
RORγT - PE
ILC2
KLRG1 - APC-Cy7
ILC1
NK Cells
CD127 - PE-Cy5
Eomes - PE-Texas Red
CD4+ Lti cells
NCR- ILC3
CD4- Lti cells
CD4 - AF700
CCR6 - APC

Figure S3. Certain subsets of ILCs are minimally impacted if at all with treatment of TACR1 antagonist. Mice were infected with *C. rodentium* or LB and treated with vehicle or CP96345 orally. Ten days post infection, MLN CD3+ CD4+ T-cells were assessed for their expression of FoxP3 via intracellular cytokine staining **(A)**. Three days post infection, colonic ILC1 **(B)**, ILC2 **(C)**, NK cells **(D)**, NCR+ ILC3 **(E)**, NCR- ILC3 **(F)**, CD4- Lti **(G)**, and CD4+ Lti **(H)** cells were quantified by frequency of live. Gating strategy for each of these populations **(I)**. Results are from individual mice, mean ± SEM, * = P ≤0.05, ** = P ≤0.01, *** = P ≤0.001 compared to uninfected controls of the same treatment and # = P ≤0.05, ## = P ≤0.01, ### = P ≤0.001 compared between treatment groups. One-way ANOVA with Tukey's post-hoc test was used. Lamina propria (LP), Luria broth (LB), *C. rodentium* (*C. rod*), innate lymphoid cell (ILC), natural killer (NK), natural cytotoxicity receptor (NCR), lymphoid tissue inducer (LTi).

Figure S4

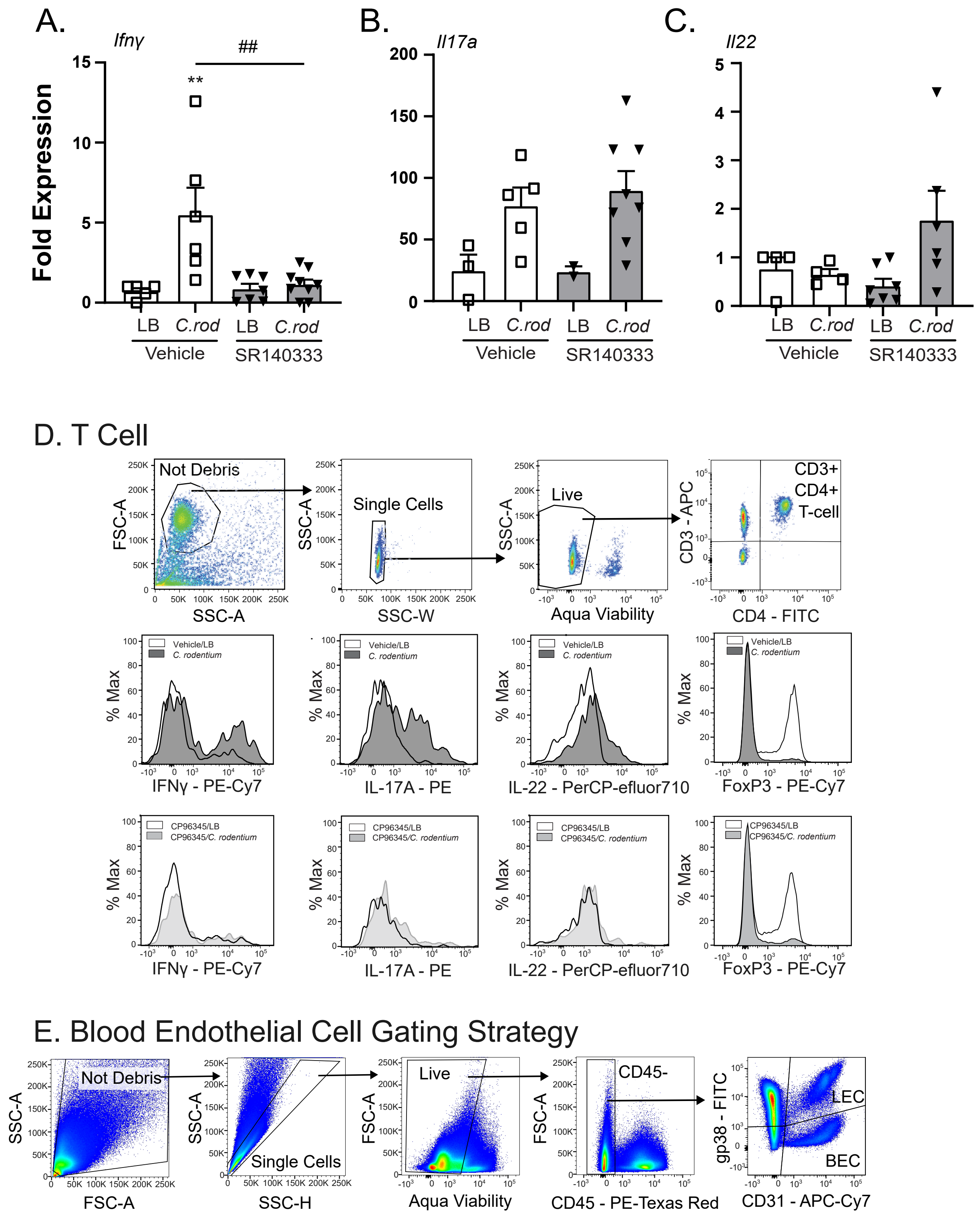

A.
Ifnγ
Fold Expression
**
##
B.
Il17a
C.
Il22
LB
C.rod
Vehicle
SR140333
D. T Cell
Not Debris
Single Cells
Live
CD3+
CD4+
T-cell
FSC-A
SSC-A
SSC-W
Aqua Viability
CD3 - APC
CD4 - FITC
Vehicle/LB
C. rodentium
% Max
IFNγ - PE-Cy7
IL-17A - PE
IL-22 - PerCP-efluor710
FoxP3 - PE-Cy7
CP96345/LB
CP96345/C. rodentium
E. Blood Endothelial Cell Gating Strategy
Not Debris
Single Cells
Live
CD45-
LEC
BEC
SSC-H
CD45 - PE-Texas Red
gp38 - FITC
CD31 - APC-Cy7

Figure S4. IFNγ expression is decreased in mice treated with different TACR1 antagonist through a separate drug delivery route & lamina propria flow cytometry gating strategies. Mice infected with *C. rodentium* or LB 10 dpi and treated with 1 mg/kg SR140333 i.p. or vehicle were assessed for their mRNA expression of *Ifnγ* **(A)**, *Il17a* **(B)**, and *Il22* **(C)** in distal colonic tissue. T-cell **(D)** and BEC **(E)** gating strategy used in Figure 2. **(A-C)** Results are from individual mice, mean ± SEM, * = P ≤0.05, ** = P ≤0.01, *** = P ≤0.001 compared to uninfected controls of the same treatment and # = P ≤0.05, ## = P ≤0.01, ### = P ≤0.001 compared between treatment groups. One-way ANOVA with Tukey's post-hoc test was used. Luria broth (LB), *C. rodentium* (*C. rod*).

Figure S5

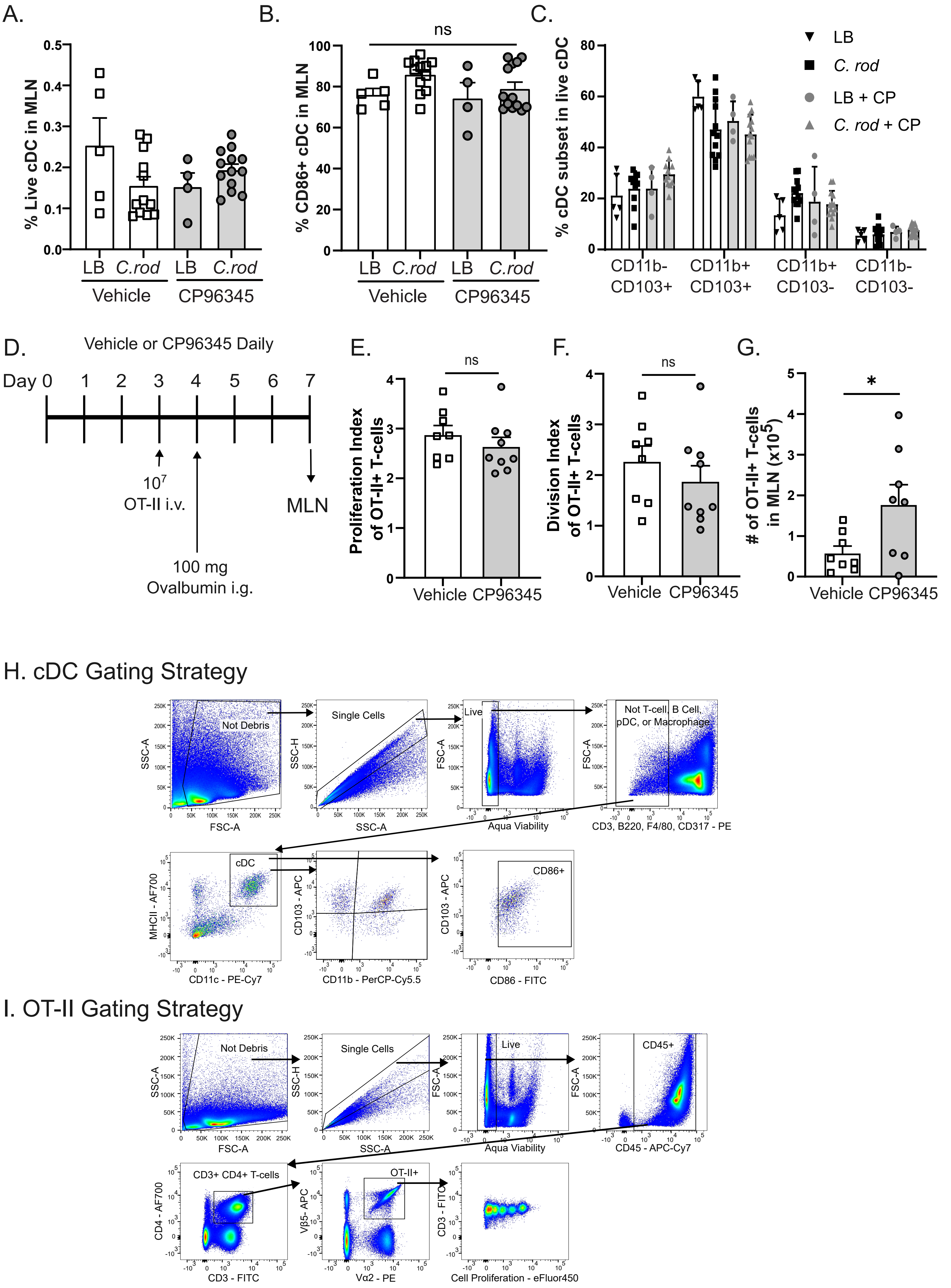

A.
% Live cDC in MLN
LB
C.rod
LB
C.rod
Vehicle
CP96345
B.
ns
% CD86+ cDC in MLN
Vehicle
CP96345
C.
% cDC subset in live cDC
LB
C. rod
LB + CP
C. rod + CP
CD11b- CD103+
CD11b+ CD103+
CD11b+ CD103-
CD11b- CD103-
D.
Vehicle or CP96345 Daily
Day 0 1 2 3 4 5 6 7
10^7 OT-II i.v.
100 mg Ovalbumin i.g.
MLN
E.
ns
Proliferation Index of OT-II+ T-cells
Vehicle
CP96345
F.
ns
Division Index of OT-II+ T-cells
Vehicle
CP96345
G.
*
# of OT-II+ T-cells in MLN (x10^5)
Vehicle
CP96345
H. cDC Gating Strategy
Not Debris
Single Cells
Live
Not T-cell, B Cell, pDC, or Macrophage
FSC-A
SSC-A
Aqua Viability
CD3, B220, F4/80, CD317 - PE
cDC
CD86+
MHCII - AF700
CD103 - APC
CD11c - PE-Cy7
CD11b - PerCP-Cy5.5
CD86 - FITC
I. OT-II Gating Strategy
Not Debris
Single Cells
Live
CD45+
CD45 - APC-Cy7
CD3+ CD4+ T-cells
OT-II+
CD4 - AF700
Vβ5- APC
CD3 - FITC
Vα2 - PE
Cell Proliferation - eFluor450

Figure S5. Conventional dendritic cell function and antigen specific T-cell proliferation is not reduced by TACR1 antagonism. Conventional dendritic cells (cDC) in MLN were analyzed from mice 10 dpi with *C. rodentium* or LB and treated with CP96345 or vehicle. The frequency of live cDC **(A)**, cDC expressing CD86 **(B)**, and different cDC subsets shown by their expression of CD11b and CD103 **(C)** were analyzed by flow cytometry. **(D)** WT mice were pre-treated for 3 days and continually treated for 4 days after with CP96345 or vehicle orally before having Cell Proliferation dyed CD4+ OT-II cells adoptively transferred. One day later, a bolus of ovalbumin was administered orally. Three days after that, MLN was analyzed for CD4+ OT-II+ T-cells. Proliferation index **(E)**, division index **(F)**, and total cell number of OT-II+ T-cells in the MLN **(G)** were quantified. Gating strategies for cDC **(H)** and OT-II T-cells **(I)**. Results are from individual mice, mean ± SEM, * = P ≤0.05, ** = P ≤0.01, *** = P ≤0.001. One-way ANOVA with Tukey's post-hoc test was used. Luria broth (LB), *C. rodentium* (*C. rod*), CP96345 (CP).

Figure S6

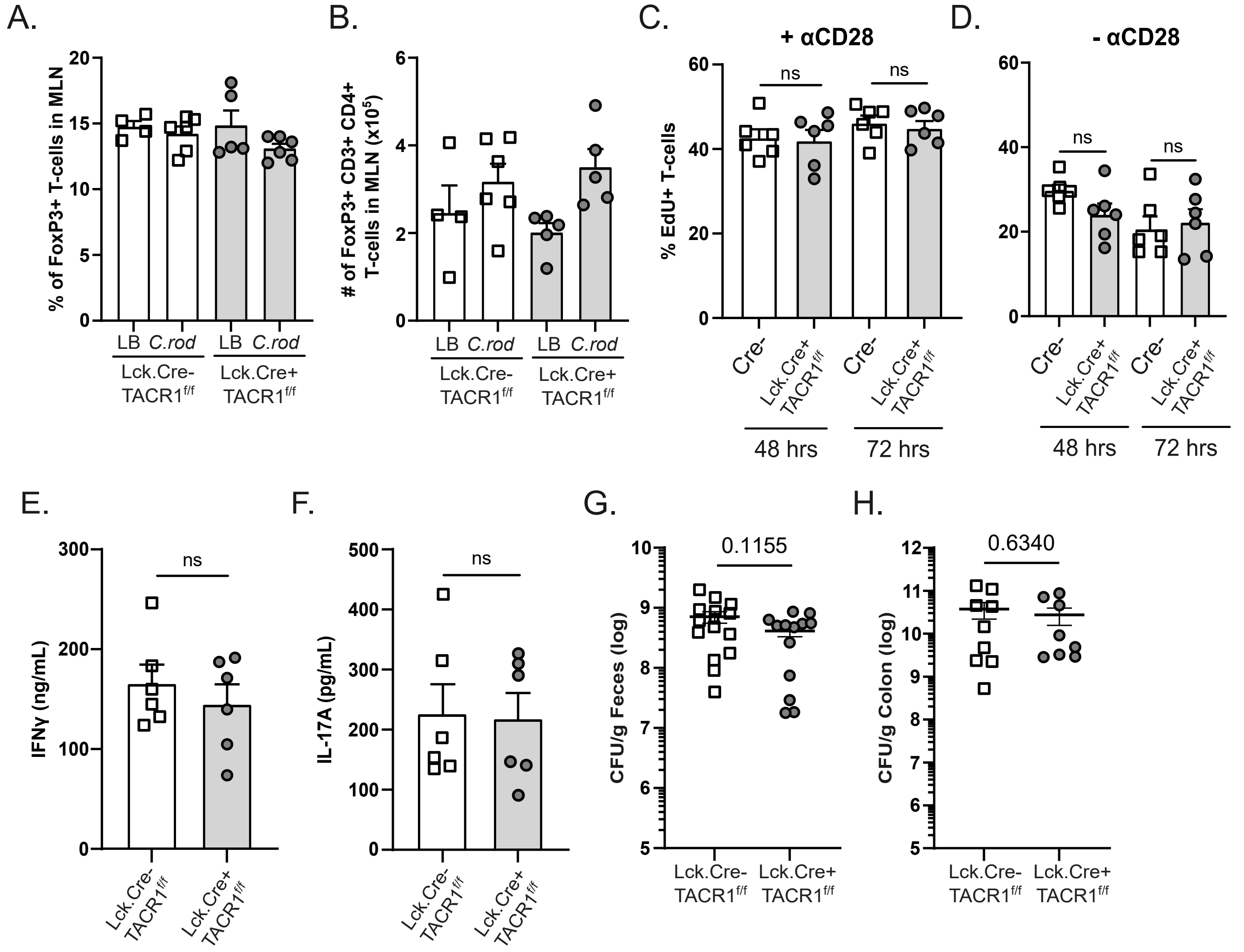

A.
% of FoxP3+ T-cells in MLN
LB C.rod LB C.rod
Lck.Cre- TACR1f/f
Lck.Cre+ TACR1f/f
B.
# of FoxP3+ CD3+ CD4+ T-cells in MLN (x10^5)
C.
+ αCD28
% EdU+ T-cells
ns
Cre-
Lck.Cre+ TACR1f/f
48 hrs
72 hrs
D.
- αCD28
E.
IFNγ (ng/mL)
F.
IL-17A (pg/mL)
G.
0.1155
CFU/g Feces (log)
H.
0.6340
CFU/g Colon (log)

Figure S6. TACR1 deletion on T-cells does not cause significant defects of the T-cell's ability to proliferate or produce cytokines *in vitro*. MLN were analyzed for FoxP3 expressing CD4+ T-cells in Lck.Cre+ TACR1$^{f/f}$ mice and their Lck.Cre- TACR1$^{f/f}$ littermates 10 dpi of *C. rodentium* infection or uninfected (LB) controls. Frequency of FoxP3+ in CD3+ CD4+ T-cells **(A)**, and total number of FoxP3+ CD3+ CD4+ T-cells in the MLN **(B)** were quantified. Splenic and lymph node CD4+ T-cells from Lck.Cre+ TACR1$^{f/f}$ mice or Lck.Cre- TACR1$^{f/f}$ littermates were cultured *in vitro* with anti-CD3 and anti-CD28 **(C)** or not **(D)** and assessed for their proliferation with EdU pulse for 2 hours or cultured in the presence of T-cell subset skewing cytokine cocktails to produce IFNγ **(E)** or IL-17A **(F)**. Supernatant of these cultures were quantified by ELISA. Fecal **(G)** and colonic **(H)** *C. rodentium* bacterial burden was quantified in Lck.Cre+ TACR1$^{f/f}$ mice and their Lck.Cre- TACR1$^{f/f}$ littermates 10 dpi. Results are from individual mice **(A-B & G-H)** or from individual mice averaged between 2-5 technical replicates **(C-F)**, mean ± SEM, * = P ≤0.05, ** = P ≤0.01, *** = P ≤0.001. One-way ANOVA with Tukey's post-hoc test was used. Luria broth (LB), *C. rodentium* (*C. rod*).

Table S1. List of primers used for qPCR

Target	Forward 5'-3'	Reverse 5'-3'
Il-1β	CTGTGACTCAT GGGATGATGATG	CGGAGCCTGTAGTGCAGTTG
Il-6	TAGTCCTTCCTACCCCAATTTCC	TTGGTCCTTAGCCACTCCTTC
Il-17a	TTTAACTCCCTTGGCGCAAAA	CTTTCCCTCCGCATTGACAC
Il-22	ATGAGTTTTTCCCTTATGGGGAC	CTGGAAGTTGGACACCTCAA
Ifnγ	GCCACGGCACAGTCATTGA	TGCTGATGGCCTGATTGTCTT
Tnfa	CCCTCACACTCAGATCATCTTCT	GCTACGACGTGGGCTACAG
Nos2	GTTCTCAGCCCAACAATACAAGA'	GTGGACGGGTCGATGTCAC
Chi3l3	CTCTGTTCAGCTATTGGACGC	CGGAATTTCTGGGATTCAGCTTC
Regiiiγ	CCTCAGGACATCTTGTGTC	TCCACCTCTGTTGGGTTCA
β-actin	GGCTGTATTCCCCTCCATCG	CCAGTTGGTAACAATGCCATGT
Tacr1	CTCCACCAACACTTCTGAGTC	TCACCACTGTATTGAATGCAGC
espA	ATCTTACGGCTGAGTTAAGCG	CGGCTATTATCCACCGTCG
espB	TCTCATCTGTCCTGGGGATT	ACTTCAGAGGCGGTATTGAC
ler	AATATACCTGATGGTCTTG	TTCTTCCATTCAATAATGCTTCTT
rrsA	AGGCCTTCGGGTTGTAAAGT	ATTCCGATTAACGCTTGCAC
Atoh1	GAGTGGGCTGAGGTAAAAGAGT	GGTCGGTGCTATCCAGGAG
H2ab1	AGCCCCATCACTGTGGAGT	GATGCCGCTCAACATCTTGC

Table S2. Antibodies used for confocal

**Target**	**Batch No**	**Host**	**Source**	**Catalog No**	**Dilution**
CD3	159344	Rat	Bio-Rad	CD3-12	1:200
CDH1	14	Mouse	ECM Bioscience	CP1921	1:300
Ki67	MK167	Rabbit	LS Bio	LS-C141898	1:600
C. koseri (cross-reactive *to C. rodentium)*	GR324195-4	rabbit	Abcam	Ab37056	1:500
**Target**		**Conjugate**	**Source**	**Catalog No**	**Dilution**
Streptavidin	2170983	Alexa Fluor 488	Thermofisher	S32354	1:200
Streptavidin	2179341	Alexa Fluor 647	Thermofisher	S32357	1:200
Goat Anti-rabbit	1616933	Alexa Fluor 488	Thermofisher	A32732	1:200
Donkey Anti-rat	1744717	Alexa Fluor 488	Thermofisher	A21208	1:200

Table S3. Flow cytometry antibodies

Antibody Target	Manufacture & Clone	Catalog number
CD3	Tonbo Biosciences 145-2C11	20-0031
CD4	BD Biosciences RM4-5	553047
CD45	Invitrogen 30-F11	48-0451-82
IFNγ	Invitrogen XMG1.2	25-7311-82
IL-17A	BD Biosciences TC11-18H10	561020
IL-22	Invitrogen 1HBPWSR	46-7221-82
CD31	Biolegend MEC13.3	102533
gp38	Biolegend 8.1.1	127405
ICAM-1	Biolegend YN1/1.7.4	116114
VCAM-1	Biolegend 429(MVCAM.A)	105719
MAdCAM-1	Biolegend MECA-367	120710
Fixable Live/Dead Aqua	ThermoFisher	L34957
Vα2	Invitrogen B20.1	12-5812
Vβ5	Invitrogen MR9-4	17-5796
CD11c	Invitrogen N418	25-0114
MHCII	Invitrogen M5/114.15.2	56-5321
CD103	BD Biosciences M290	562772
CD11b	Invitrogen M1/70	45-0112
CD86	BD Biosciences GL1	11-0862
NKp46	Invitrogen 29A1.4	25-3351
RORyT	Invitrogen AFKJS-9	12-6988

KLRG1	Invitrogen 2F1	47-5893
Eomes	Invitrogen Dan11mag	61-4875
CD127	Invitrogen A7R34	15-1271
CCR6	R&D Systems 140706	FAB590A
CD19	BD Biosciences ID3	561740
Gr-1	Invitrogen RB6-8C5	11-5931
F4/80	Invitrogen BM8	53-4801
CD5	BD Biosciences Ly-1	553020
TCR β	BD Biosciences H57-597	553170
TCR yδ	BD Biosciences GL3	561996
FoxP3	Invitrogen FJK-16s	25-5773
B220	eBioscience RA3-6B2	12-0452
CD317	eBioscience BST2	12-3172
